# Supplementary material for: Supervised dimensionality reduction for big data
Source: Nat Commun. 2021 May 17;12:2872. doi: 10.1038/s41467-021-23102-2 (PMC8129083; doi:10.1038/s41467-021-23102-2)
Supplement: Supplementary file 1 — Supplementary Information [file 41467_2021_23102_MOESM1_ESM.pdf]

Supplementary Information: Supervised Dimensionality Reduction for Big Data

Joshua T. Vogelstein[1*†], Eric W. Bridgeford[1*], Minh Tang[1], Da Zheng[1], Christopher Douville[1], Randal Burns[1], Mauro Maggioni[1]

[1] Johns Hopkins University 3900 N Charles Street, Baltimore, MD 21218, *These authors contributed equally, †Corresponding Author, jovo@jhu.edu

Supplementary Note 1 Theoretical Background

1.1 The Classification Problem

Let $(\boldsymbol{X}, Y)$ be a pair of random variables, jointly sampled from $F := F_{\boldsymbol{X},Y} = F_{\boldsymbol{X}|Y}F_Y$, with density denoted $f_{\boldsymbol{X},Y}$. Let $\boldsymbol{X}$ be a multivariate vector-valued random variable, such that its realizations live in p dimensional Euclidean space, $\boldsymbol{x} \in \mathbb{R}^p$. Let Y be a categorical random variable, whose realizations are discrete, $y \in \{0, \ldots C-1\}$. The goal of a classification problem is to find a function $g(\boldsymbol{x})$ such that its output tends to be the true class label y:

$$g^*(\boldsymbol{x}) := \operatorname*{argmax}_{g \in \mathcal{G}} \mathbb{P}[g(\boldsymbol{x}) = y]. \tag{1}$$

When the joint distribution of the data is known, then the Bayes optimal solution is:

$$g^*(\boldsymbol{x}) := \operatorname*{argmax}_y f_{y,\boldsymbol{x}} = \operatorname*{argmax}_y f_{\boldsymbol{x}|y} f_y = \operatorname*{argmax}_y \{\log f_{\boldsymbol{x}|y} + \log f_y\} \tag{2}$$

Denote expected misclassification rate of classifier g for a given joint distribution F,

$$L_g^F := \mathbb{E}[g(\boldsymbol{x}) \neq y] := \int \mathbb{P}[g(\boldsymbol{x}) \neq y] f_{\boldsymbol{x},y} d\boldsymbol{x} dy, \tag{3}$$

where $\mathbb{E}$ is the expectation, which in this case, is with respect to F_{XY}. For brevity, we often simply write L_g, and we define $L_* := L_{g^*}$.

1.2 Linear Discriminant Analysis (LDA)

Linear Discriminant Analysis (LDA) is an approach to classification that uses a linear function of the first two moments of the distribution of the data. More specifically, let $\boldsymbol{\mu}_j = \mathbb{E}[F_{X|Y=j}]$ denote the class conditional mean, and let $\boldsymbol{\Sigma} = \mathbb{E}[F_X^2]$ denote the joint covariance matrix, and the class priors are $\pi_j = \mathbb{P}[Y = j]$. Using this notation, we can define the LDA classifier:

$$g_{\text{LDA}}(\boldsymbol{x}) := \operatorname*{argmin}_y \left[\frac{1}{2}(\boldsymbol{x} - \boldsymbol{\mu}_y)^\mathsf{T} \boldsymbol{\Sigma}^{-1}(\boldsymbol{x} - \boldsymbol{\mu}_y) - \log \pi_y\right], \tag{4}$$

Let L_{LDA}^F be the expected misclassification rate of the above classifier for distribution F. Assuming equal class prior and centered means, re-arranging a bit, we obtain

$$g_{\text{LDA}}(\boldsymbol{x}) := \operatorname*{argmin}_y \boldsymbol{x}^\mathsf{T} \boldsymbol{\Sigma}^{-1} \boldsymbol{\mu}_y. \tag{5}$$

In words, the LDA classifier chooses the class that maximizes the magnitude of the projection of an input vector $\boldsymbol{x}$ onto $\boldsymbol{\Sigma}^{-1}\boldsymbol{\mu}_y$. When there are only two classes, letting $\boldsymbol{\delta} = \boldsymbol{\mu}_0 - \boldsymbol{\mu}_1$, the above further simplifies to

$$g_{2\mathtt{LDA}}(\boldsymbol{x}) := \mathbb{I}\{\boldsymbol{x}^\mathsf{T}\boldsymbol{\Sigma}^{-1}\boldsymbol{\delta} > 0\}. \tag{6}$$

Note that the equal class prior and centered means assumptions merely changes the threshold constant from 0 to some other constant.

1.3 LDA Model

A statistical model is a family of distributions indexed by a parameter $\boldsymbol{\theta} \in \boldsymbol{\Theta}$, $\mathcal{F}_{\boldsymbol{\theta}} = \{F_{\boldsymbol{\theta}} : \boldsymbol{\theta} \in \boldsymbol{\Theta}\}$. Consider the special case of the above where $F_{\boldsymbol{X}|Y=y}$ is a multivariate Gaussian distribution, $\mathcal{N}(\boldsymbol{\mu}_y, \boldsymbol{\Sigma})$, where each class has its own mean, but all classes have the same covariance. We refer to this model as the LDA model. Let $\boldsymbol{\theta} = (\boldsymbol{\pi}, \boldsymbol{\mu}, \boldsymbol{\Sigma})$, and let $\boldsymbol{\Theta}_{C-\mathtt{LDA}} = (\triangle_C, \mathbb{R}^{p\times C}, \mathbb{R}^{p\times p}_{\succ 0})$, where $\boldsymbol{\mu} = (\boldsymbol{\mu}_1, \ldots, \boldsymbol{\mu}_C)$, $\triangle_C$ is the C dimensional simplex, that is $\triangle_C = \{\boldsymbol{x} : x_i \geq 0 \forall i, \sum_i x_i = 1\}$, and $\mathbb{R}^{p\times p}_{\succ 0}$ is the set of positive definite $p \times p$ matrices. Denote $\mathcal{F}_{\mathtt{LDA}} = \{F_{\boldsymbol{\theta}} : \boldsymbol{\theta} \in \boldsymbol{\Theta}_{\mathtt{LDA}}\}$. The following lemma is well known [5]:

Lemma 1. $L^F_{\mathit{LDA}} = L^F_*$ *for any* $F \in \mathcal{F}_{\mathit{LDA}}$.

Supplementary Note 2 Formal Definition of LOL and Related Classifiers

Let $\boldsymbol{A} \in \mathbb{R}^{d\times p}$ be a "projection matrix", that is, a matrix that projects p-dimensional data into a d-dimensional subspace. The question that motivated this work is: what is the best projection matrix that we can estimate, to use to "pre-process" the data prior to classifying the data? Projecting the data $\boldsymbol{x}$ onto a low-dimensional subspace, and then classifying via LDA in that subspace is equivalent to redefining the parameters in the low-dimensional subspace, $\boldsymbol{\Sigma}_A = \boldsymbol{A}\boldsymbol{\Sigma}\boldsymbol{A}^\mathsf{T} \in \mathbb{R}^{d\times d}$ and $\boldsymbol{\delta}_A = \boldsymbol{A}\boldsymbol{\delta} \in \mathbb{R}^d$, and then using $g_{\mathtt{LDA}}$ in the low-dimensional space. When $C = 2$, $\pi_0 = \pi_1$, and $(\mu_0 + \mu_1)/2 = \boldsymbol{0}$, this amounts to:

$$g^d_A(x) := \mathbb{I}\{(\boldsymbol{A}\boldsymbol{x})^\mathsf{T}\boldsymbol{\Sigma}_A^{-1}\boldsymbol{\delta}_A > 0\}, \text{ where } \boldsymbol{A} \in \mathbb{R}^{d\times p}. \tag{7}$$

Let $L^d_A := \int \mathbb{P}[g^d_A(\boldsymbol{x}) = y] f_{\boldsymbol{x},y} d\boldsymbol{x} dy$. Our goal therefore is to be able to choose A for a given parameter setting $\boldsymbol{\theta} = (\boldsymbol{\pi}, \boldsymbol{\delta}, \boldsymbol{\Sigma})$, such that L_A is as small as possible (note that L_A will never be smaller than L_*).

In the naive case where $(\boldsymbol{\Sigma}_A, \boldsymbol{\delta}_A)$ are known, we seek to solve the following linear optimization problem:

$$\begin{aligned} \underset{\boldsymbol{A}}{\text{minimize}} \quad & \mathbb{E}[\mathbb{I}\{\boldsymbol{x}^\mathsf{T}\boldsymbol{A}^\mathsf{T}\boldsymbol{\Sigma}_A^{-1}\boldsymbol{\delta}_A > 0\} \neq y] \\ \text{subject to} \quad & \boldsymbol{A} \in \mathbb{R}^{d\times p}. \end{aligned} \tag{8}$$

When $(\boldsymbol{\Sigma}_A, \boldsymbol{\delta}_A)$ are not known, however, the optimization problem becomes non-convex. With $\boldsymbol{\Sigma}_A$ and $\boldsymbol{\delta}_A$ as above:

$$\begin{aligned} \underset{\boldsymbol{A},\boldsymbol{\Sigma},\boldsymbol{\delta}}{\text{minimize}} \quad & \mathbb{E}[\mathbb{I}\{\boldsymbol{x}^\mathsf{T}\boldsymbol{A}^\mathsf{T}\boldsymbol{\Sigma}_A^{-1}\boldsymbol{\delta}_A > 0\} \neq y] \\ \text{subject to} \quad & \boldsymbol{A} \in \mathbb{R}^{d\times p}. \end{aligned} \tag{9}$$

While there are numerous approaches to solve related convex optimization problems through various sets of assumptions [10, 15], we do not consider such techniques in this manuscript theoretically. This

is because assuming either a structure for $\boldsymbol{\Sigma}_A$ or $\boldsymbol{\delta}_A$ presupposes an understanding of the properties of the feature space for wide data, which is often unsuitable if the dataset is large or has considerable complexity.

Let $\mathcal{A} = \{\boldsymbol{A} : \boldsymbol{A} \in \mathbb{R}^{k \times p}, k \leq p\}$, $\mathcal{A}^d = \{\boldsymbol{A} : \boldsymbol{A} \in \mathbb{R}^{d \times p}\}$, and let $\mathcal{A}_* \subset \mathcal{A}$ be the set of $\boldsymbol{A}$ that minimizes Supp. Eq. (9), and let $\boldsymbol{A}_* \in \mathcal{A}_*$. Let $L^*_{\boldsymbol{A}} = L_{\boldsymbol{A}_*}$ be the misclassification rate for any $\boldsymbol{A} \in \mathcal{A}_*$, that is, $L^*_{\boldsymbol{A}}$ is the Bayes optimal misclassification rate for the classifier that composes $\boldsymbol{A}$ with LDA.

In our opinion, Supp. Eq. (9) is the simplest supervised manifold learning problem there is: a two-class classification problem, where the data are multivariate Gaussians with shared, unknown covariances, the manifold is linear, and the classification is done via LDA. Nonetheless, solving Supp. Eq. (9) is difficult, because we do not know how to evaluate the integral analytically, and we do not know any algorithms that are guaranteed to find the global optimum in finite time. We proceed by studying a few natural choices for $\boldsymbol{A}$.

2.1 Bayes Optimal Projection

Lemma 2. $\boldsymbol{\delta}^{\mathsf{T}}\boldsymbol{\Sigma}^{-1} \in \mathcal{A}_*$

Proof. Let $\boldsymbol{B} = (\boldsymbol{\Sigma}^{-1}\boldsymbol{\delta})^{\mathsf{T}} = \boldsymbol{\delta}^{\mathsf{T}}(\boldsymbol{\Sigma}^{-1})^{\mathsf{T}} = \boldsymbol{\delta}^{\mathsf{T}}\boldsymbol{\Sigma}^{-1}$, so that $\boldsymbol{B}^{\mathsf{T}} = \boldsymbol{\Sigma}^{-1}\boldsymbol{\delta}$, and plugging this in to Supp. Eq. (7). By the above, and noting the symmetry and invertibility of $\boldsymbol{\Sigma}$:

$$
\begin{aligned}
\boldsymbol{\Sigma}_B &= \boldsymbol{B}\boldsymbol{\Sigma}\boldsymbol{B}^{\mathsf{T}} = \boldsymbol{\delta}^{\mathsf{T}}\boldsymbol{\Sigma}^{-1}\boldsymbol{\Sigma}(\boldsymbol{\delta}^{\mathsf{T}}\boldsymbol{\Sigma}^{-1})^{\mathsf{T}} \\
&= \boldsymbol{\delta}^{\mathsf{T}}\boldsymbol{\Sigma}^{-1}\boldsymbol{\Sigma}\boldsymbol{\Sigma}^{-1}\boldsymbol{\delta} = \boldsymbol{\delta}^{\mathsf{T}}\boldsymbol{\Sigma}^{-1}\boldsymbol{\delta} \\
\Rightarrow \boldsymbol{\Sigma}_B^{-1} &= \boldsymbol{\delta}^{-1}\boldsymbol{\Sigma}\boldsymbol{\delta}^{\mathsf{T}-1} \\
\boldsymbol{\delta}_B &= \boldsymbol{B}\boldsymbol{\delta} = \boldsymbol{\delta}^{\mathsf{T}}\boldsymbol{\Sigma}^{-1}\boldsymbol{\delta}
\end{aligned}
$$

We obtain:

$$
\begin{aligned}
g_B(x) &= \mathbb{I}\{\boldsymbol{x}^{\mathsf{T}}\boldsymbol{B}^{\mathsf{T}}\boldsymbol{\Sigma}_B^{-1}\boldsymbol{\delta}_B > 0\} \\
&= \mathbb{I}\{\boldsymbol{x}^{\mathsf{T}}(\boldsymbol{\Sigma}^{-1}\boldsymbol{\delta})(\boldsymbol{\Sigma}_B^{-1}\boldsymbol{\delta}_B) > 0\} && \text{plugging in } \boldsymbol{B} \\
&= \mathbb{I}\{\boldsymbol{x}^{\mathsf{T}}(\boldsymbol{\Sigma}^{-1}\boldsymbol{\delta})(\boldsymbol{\delta}^{-1}\boldsymbol{\Sigma}\boldsymbol{\delta}^{\mathsf{T}-1}\boldsymbol{\delta}^{\mathsf{T}}\boldsymbol{\Sigma}^{-1}\boldsymbol{\delta}) > 0\} && \text{plug in } \boldsymbol{\Sigma}_B, \boldsymbol{\delta}_B \text{ from above} \\
&= \mathbb{I}\{\boldsymbol{x}^{\mathsf{T}}\boldsymbol{\Sigma}^{-1}\boldsymbol{\delta} > 0\}
\end{aligned}
$$

In other words, letting $\boldsymbol{B}$ be the Bayes optimal projection recovers the Bayes classifier, as it should. Or, more formally, for any $F \in \mathcal{F}_{\text{LDA}}$, $L_{\boldsymbol{\delta}^{\mathsf{T}}\boldsymbol{\Sigma}^{-1}} = L_*$. □

2.2 Principle Components Analysis (PCA) Projection

Principle Components Analysis (PCA) finds the directions of maximal variance in a dataset. PCA is closely related to eigendecompositions and singular value decompositions (SVD). In particular, the top left singular vector of a matrix $\boldsymbol{X} \in \mathbb{R}^{p \times n}$, whose columns are centered, is the eigenvector with the largest eigenvalue of the centered covariance matrix $\boldsymbol{X}\boldsymbol{X}^{\mathsf{T}}$. SVD enables one to estimate this eigenvector without ever forming the outer product matrix, because SVD factorizes a matrix $\boldsymbol{X}$ into $\boldsymbol{U}\boldsymbol{S}\boldsymbol{V}^{\mathsf{T}}$, where $\boldsymbol{U}$ and $\boldsymbol{V}$ are orthonormal $p \times n$ matrices, and $\boldsymbol{S}$ is a diagonal matrix, whose diagonal values are decreasing, $s_1 \geq s_2 \geq \cdots > s_n$. Defining $\boldsymbol{U} = [\boldsymbol{u}_1, \boldsymbol{u}_2, \ldots, \boldsymbol{u}_n]$, where each $\boldsymbol{u}_i \in \mathbb{R}^p$, then $\boldsymbol{u}_i$ is the ith eigenvector, and s_i is the square root of the ith eigenvalue of $\boldsymbol{X}\boldsymbol{X}^{\mathsf{T}}$. Let $\boldsymbol{A}_d^{\text{PCA}} =$

$[\boldsymbol{u}_1, \ldots, \boldsymbol{u}_d]$ be the truncated PCA orthonormal matrix, and let $\boldsymbol{I}_{d\times p}$ denote a $d \times p$ dimensional identity matrix.

The PCA matrix is perhaps the most obvious choice of an orthonormal matrix for several reasons. First, truncated PCA minimizes the squared error loss between the original data matrix and all possible rank d representations:

$$\underset{A\in\mathbb{R}^{d\times p}}{\operatorname{argmin}} \left\|\boldsymbol{X} - \boldsymbol{A}^T\boldsymbol{A}\right\|_F^2. \tag{10}$$

Second, the ubiquity of PCA has led to a large number of highly optimized numerical libraries for computing PCA (for example, LAPACK [2]).

In this supervised setting, we consider two different variants of PCA, each based on centering the data differently. For the first one, which we refer to as "pooled PCA" (or just PCA for brevity), we center the data by subtracting the "pooled mean" from each sample, that is, we let $\widetilde{\boldsymbol{x}}_i = \boldsymbol{x} - \boldsymbol{\mu}$, where $\boldsymbol{\mu} = \mathbb{E}[\boldsymbol{x}]$. For the second, which we refer to as "class conditional PCA", we center the data by subtracting the "class-conditional mean" from each sample, that is, we let $\widetilde{\boldsymbol{x}}_i = \boldsymbol{x} - \boldsymbol{\mu}_y$, where $\boldsymbol{\mu}_y = \mathbb{E}[\boldsymbol{x}|Y = y]$.

Notationally, let $\boldsymbol{U}_d = [\boldsymbol{u}_1, \ldots, \boldsymbol{u}_d] \in \mathbb{R}^{p\times d}$, and note that $\boldsymbol{U}_d^\mathsf{T}\boldsymbol{U}_d = \boldsymbol{I}_{d\times d}$ and $\boldsymbol{U}_d\boldsymbol{U}_d^\mathsf{T} = \boldsymbol{I}_{p\times p}$. Similarly, let $\boldsymbol{U}\boldsymbol{S}\boldsymbol{U}^\mathsf{T} = \boldsymbol{\Sigma}$, and $\boldsymbol{U}\boldsymbol{S}^{-1}\boldsymbol{U}^\mathsf{T} = \boldsymbol{\Sigma}^{-1}$. Let $\boldsymbol{S}_d$ be the matrix whose diagonal entries are the eigenvalues, up to the dth one, that is $\boldsymbol{S}_d(i,j) = s_i$ for $i = j \le d$ and zero otherwise. Similarly, $\boldsymbol{\Sigma}_d = \boldsymbol{U}\boldsymbol{S}_d\boldsymbol{U}^\mathsf{T} = \boldsymbol{U}_d\boldsymbol{S}_d\boldsymbol{U}_d^\mathsf{T}$. Reduced-rank LDA (rrLDA) is a regularized LDA algorithm. Specifically, rather than using the full rank covariance matrix, it uses a rank-d approximation. Formally, let $g_{\text{LDA}} := \mathbb{I}\{x\boldsymbol{\Sigma}^{-1}\boldsymbol{\delta} > 0\}$ be the LDA classifier, and let $g_{\text{LDA}}^d := \mathbb{I}\{x\boldsymbol{\Sigma}_d^{-1}\boldsymbol{\delta} > 0\}$ be the regularized LDA classifier, that is, the LDA classifier where the the bottom $p - d$ eigenvalues of the covariance matrix are set to zero.

Lemma 3. *Using class-conditional PCA to pre-process the data, then using LDA on the projected data, is equivalent to rrLDA.*

Proof. Plugging $\boldsymbol{U}_d$ into Eq. (7) for $\boldsymbol{A}$, and considering only the left side of the operand, we have

$$\begin{aligned}
(\boldsymbol{A}\boldsymbol{x})^\mathsf{T}\boldsymbol{\Sigma}_A^{-1}\boldsymbol{\delta}_A &= \boldsymbol{x}^\mathsf{T}\boldsymbol{A}^\mathsf{T}\boldsymbol{A}\boldsymbol{\Sigma}^{-1}\boldsymbol{A}^\mathsf{T}\boldsymbol{A}\boldsymbol{\delta}, \\
&= \boldsymbol{x}^\mathsf{T}\boldsymbol{U}_d\boldsymbol{U}_d^\mathsf{T}\boldsymbol{\Sigma}^{-1}\boldsymbol{U}_d\boldsymbol{U}_d^\mathsf{T}\boldsymbol{\delta}, \\
&= \boldsymbol{x}^\mathsf{T}\boldsymbol{U}_d\boldsymbol{U}_d^\mathsf{T}\boldsymbol{U}\boldsymbol{S}^{-1}\boldsymbol{U}^\mathsf{T}\boldsymbol{U}_d\boldsymbol{U}_d^\mathsf{T}\boldsymbol{\delta}, \\
&= \boldsymbol{x}^\mathsf{T}\boldsymbol{U}_d\boldsymbol{I}_{d\times p}\boldsymbol{S}^{-1}\boldsymbol{I}_{p\times d}\boldsymbol{U}_d^\mathsf{T}\boldsymbol{\delta}, \\
&= \boldsymbol{x}^\mathsf{T}\boldsymbol{U}_d\boldsymbol{S}_d^{-1}\boldsymbol{U}_d^\mathsf{T}\boldsymbol{\delta}, \\
&= \boldsymbol{x}^\mathsf{T}\boldsymbol{\Sigma}_d^{-1}\boldsymbol{\delta}.
\end{aligned}$$

□

The implication of this lemma is that if one desires to implement rrLDA, rather than first learning the eigenvectors and then learning LDA, one can instead directly implement regularized LDA by setting the bottom $p - d$ eigenvalues to zero. This latter approach removes the requirement to run SVD twice, therefore reducing the computational burden as well as the possibility of numerical instability issues. We therefore refer to the projection composed of d eigenvectors of the class-conditionally centered covariance matrices, $\boldsymbol{A}_{\text{LDA}}^d$.

2.3 Linear Optimal Low-Rank (LOL) Projection

The basic idea of LOL is to use both $\boldsymbol{\delta}$ and the top d eigenvectors of the class-conditionally centered covariance. When there are only two classes, $\boldsymbol{\delta} = \boldsymbol{\mu}_0 - \boldsymbol{\mu}_1$. When there are $C > 2$ classes, there are $\binom{C}{2} = \frac{C!}{2(C-2)!}$ pairwise combinations, $\boldsymbol{\delta}_{ij} = \boldsymbol{\mu}_i - \boldsymbol{\mu}_j$ for all $i \neq j$. However, since $\binom{C}{2}$ is nearly C^2, when C is large, this would mean incorporating many mean difference vectors. Note that $[\boldsymbol{\delta}_{1,2}, \boldsymbol{\delta}_{1,3}, \ldots, \boldsymbol{\delta}_{C-1,C}]$ is in fact a rank $C-1$ matrix, because it is a linear function of the C different means. Therefore, we only need $C-1$ differences to span the space of all pairwise differences. To mitigate numerical instability issues, we adopt the following convention. For each class, estimate the expected mean and the number of samples per class, $\boldsymbol{\mu}_c$ and π_c. Sort the means in order of decreasing π_c, so that $\pi_{(1)} > \pi_{(2)} > \cdots > \pi_{(C)}$. Then, subtract $\boldsymbol{\mu}_{(1)}$ from all other means: $\boldsymbol{\delta}_i = \boldsymbol{\mu}_{(1)} - \boldsymbol{\mu}_{(i)}$, for $i = 2, \ldots, C$. Finally, $\boldsymbol{\delta} = [\boldsymbol{\delta}_2, \ldots, \boldsymbol{\delta}_C]$.

Given $\boldsymbol{\delta}$ and $\boldsymbol{A}^{d-1}_{\texttt{LDA}}$, to obtain LOL naïvely, we could simply concatenate the two, $\boldsymbol{A}^{d}_{\texttt{LOL},naive} = [\boldsymbol{\delta}, \boldsymbol{A}^{d-1}_{\texttt{LDA}}]$. Recall that eigenvectors are orthonormal. To maintain orthonormality between the eigenvectors and vectors of $\boldsymbol{\delta}$, we could easily apply Gram-Schmidt, $\boldsymbol{A}^{d}_{\texttt{LOL},naive} = \texttt{Orth}([\boldsymbol{\delta}, \boldsymbol{A}^{d-1}_{\texttt{LDA}}])$. In practice, this orthogonalization step does not matter much, so we ignore it hereafter. To ensure that $\boldsymbol{\delta}$ and $\boldsymbol{\Sigma}$ are balanced appropriately, we normalize each vector in $\boldsymbol{\delta}$ to have norm unity. Formally, let $\widetilde{\boldsymbol{\delta}}_j = \boldsymbol{\delta}_j / \|\boldsymbol{\delta}_j\|$, where $\boldsymbol{\delta}_j$ is the jth difference of the mean vector and let $\boldsymbol{A}^{d}_{\texttt{LOL}} = [\widetilde{\boldsymbol{\delta}}, \boldsymbol{A}^{d-(C-1)}_{\texttt{LDA}}]$.

When the distribution of the data is not provided, each of the above terms must be estimated from the data. We use the maximum likelihood estimators for each, specifically:

$$n_c = \sum_{i=1}^{n} \mathbb{I}\{y_i = c\}, \tag{11}$$

$$\hat{\pi}_c = \frac{n_c}{n}, \tag{12}$$

$$\hat{\boldsymbol{\mu}} = \frac{1}{n}\sum_{i=1}^{n} \boldsymbol{x}_i, \tag{13}$$

$$\hat{\boldsymbol{\mu}}_c = \frac{1}{n_c}\sum_{i=1}^{n} \boldsymbol{x}_i \mathbb{I}\{y_i = c\}. \tag{14}$$

For completeness, below we provide pseudocode for learning the sample version of LOL. The population version does not require the estimation of the parameters.

2.4 rrLDA is rotationally invariant

For certain classification tasks, the observed dimensions (or features) have intrinsic value, e.g. when simple interpretability is desired. However, in many other contexts, interpretability is less important [3]. When the exploitation task at hand is invariant to rotations, then we have no reason to restrict our search space to be sparse in the observed dimensions. For example, we can consider sparsity in the eigenvector basis. Let $\boldsymbol{W}$ be a rotation matrix, that is $\boldsymbol{W} \in \mathcal{W} = \{\boldsymbol{W} : \boldsymbol{W}^{\mathsf{T}} = \boldsymbol{W}^{-1}$ and $\det(\boldsymbol{W}) = 1\}$. Moreover, let $\boldsymbol{W} \circ F$ denote the distribution F after transformation by an operator $\boldsymbol{W}$. For example, if $F = \mathcal{N}(\boldsymbol{\mu}, \boldsymbol{\Sigma})$ then $\boldsymbol{W} \circ F = \mathcal{N}(\boldsymbol{W}\boldsymbol{\mu}, \boldsymbol{W}\boldsymbol{\Sigma}\boldsymbol{W}^{\mathsf{T}})$.

Definition 1. *A rotationally invariant classifier has the following property:*

$$L_g^F = L_g^{W \circ F}, \qquad F \in \mathcal{F} \text{ and } W \in \mathcal{W}. \tag{15}$$

In words, the Bayes risk of using classifier g on distribution F is unchanged if F is first rotated.

Now, we can state the main lemma of this subsection: `rrLDA` is rotationally invariant.

Lemma 4. $L_{LDA}^{F} = L_{LDA}^{W\circ F}$*, for any* $F \in \mathcal{F}$*.*

Proof. `rrLDA` is in fact simply thresholding $\boldsymbol{x}^\mathsf{T}\boldsymbol{\Sigma}^{-1}\boldsymbol{\delta}$ whenever its value is larger than some constant. Thus, we can demonstrate rotational invariance by demonstrating that $\boldsymbol{x}^\mathsf{T}\boldsymbol{\Sigma}^{-1}\boldsymbol{\delta}$ is rotationally invariant.

$$
\begin{aligned}
(\boldsymbol{W}\boldsymbol{x})^\mathsf{T}(\boldsymbol{W}\boldsymbol{\Sigma}\boldsymbol{W}^\mathsf{T})^{-1}\boldsymbol{W}\boldsymbol{\delta} &= \boldsymbol{x}^\mathsf{T}\boldsymbol{W}^\mathsf{T}(\boldsymbol{W}\boldsymbol{U}\boldsymbol{S}\boldsymbol{U}^\mathsf{T}\boldsymbol{W}^\mathsf{T})^{-1}\boldsymbol{W}\boldsymbol{\delta} && \text{by substituting } \boldsymbol{U}\boldsymbol{S}\boldsymbol{U}^\mathsf{T} \text{ for } \boldsymbol{\Sigma} \\
&= \boldsymbol{x}^\mathsf{T}\boldsymbol{W}^\mathsf{T}(\widetilde{\boldsymbol{U}}\boldsymbol{S}\widetilde{\boldsymbol{U}}^\mathsf{T})^{-1}\boldsymbol{W}\boldsymbol{\delta} && \text{by letting } \widetilde{\boldsymbol{U}} = \boldsymbol{W}\boldsymbol{U} \\
&= \boldsymbol{x}^\mathsf{T}\boldsymbol{W}^\mathsf{T}(\widetilde{\boldsymbol{U}}\boldsymbol{S}^{-1}\widetilde{\boldsymbol{U}}^\mathsf{T})\boldsymbol{W}\boldsymbol{\delta} && \text{by the laws of matrix inverse} \\
&= \boldsymbol{x}^\mathsf{T}\boldsymbol{W}^\mathsf{T}\boldsymbol{W}\boldsymbol{U}\boldsymbol{S}^{-1}\boldsymbol{U}^\mathsf{T}\boldsymbol{W}^\mathsf{T}\boldsymbol{W}\boldsymbol{\delta} && \text{by un-substituting } \boldsymbol{W}\boldsymbol{U} = \widetilde{\boldsymbol{U}} \\
&= \boldsymbol{x}^\mathsf{T}\boldsymbol{U}\boldsymbol{S}^{-1}\boldsymbol{U}^\mathsf{T}\boldsymbol{\delta} && \text{because } \boldsymbol{W}^\mathsf{T}\boldsymbol{W} = \boldsymbol{I} \\
&= \boldsymbol{x}^\mathsf{T}\boldsymbol{\Sigma}^{-1}\boldsymbol{\delta} && \text{by un-substituting } \boldsymbol{U}\boldsymbol{S}^{-1}\boldsymbol{U}^\mathsf{T} = \boldsymbol{\Sigma}
\end{aligned}
$$

□

One implication of this lemma is that we can reparameterize without loss of generality. Specifically, defining $\boldsymbol{W} := \boldsymbol{U}^\mathsf{T}$ yields a change of variables: $\boldsymbol{\Sigma} \mapsto \boldsymbol{S}$ and $\boldsymbol{\delta} \mapsto \boldsymbol{U}^\mathsf{T}\boldsymbol{\delta} := \boldsymbol{\delta}''$, where $\boldsymbol{S}$ is a diagonal covariance matrix. Moreover, let $\boldsymbol{d} = (\sigma_1, \ldots, \sigma_D)^\mathsf{T}$ be the vector of eigenvalues, then $\boldsymbol{S}^{-1}\boldsymbol{\delta}' = \boldsymbol{d}^{-1} \odot \widetilde{\boldsymbol{\delta}}$, where $\odot$ is the Hadamard (entrywise) product. The `LDA` classifier may therefore be encoded by a unit vector, $\widetilde{\boldsymbol{d}} := \frac{1}{m}\boldsymbol{d}^{-1} \odot \widetilde{\boldsymbol{\delta}}'$, and its magnitude, $m := \left\|\boldsymbol{d}^{-1} \odot \widetilde{\boldsymbol{\delta}}\right\|$. This will be useful later.

2.5 Rotation of Projection Based Linear Classifiers

By a similar argument as above, one can easily show that:

$$
\begin{aligned}
(\boldsymbol{A}\boldsymbol{W}\boldsymbol{x})^\mathsf{T}(\boldsymbol{A}\boldsymbol{W}\boldsymbol{\Sigma}\boldsymbol{W}^\mathsf{T}\boldsymbol{A}^\mathsf{T})^{-1}\boldsymbol{A}\boldsymbol{W}\boldsymbol{\delta} &= \boldsymbol{x}^\mathsf{T}(\boldsymbol{W}^\mathsf{T}\boldsymbol{A}^\mathsf{T})(\boldsymbol{A}\boldsymbol{W})\boldsymbol{\Sigma}^{-1}(\boldsymbol{W}^\mathsf{T}\boldsymbol{A}^\mathsf{T})(\boldsymbol{A}\boldsymbol{W})\boldsymbol{\delta} \quad (16) \\
&= \boldsymbol{x}^\mathsf{T}\boldsymbol{Y}^\mathsf{T}\boldsymbol{Y}\boldsymbol{\Sigma}^{-1}\boldsymbol{Y}^\mathsf{T}\boldsymbol{Y}\boldsymbol{\delta} \quad (17) \\
&= \boldsymbol{x}^\mathsf{T}\boldsymbol{Z}\boldsymbol{\Sigma}^{-1}\boldsymbol{Z}^\mathsf{T}\boldsymbol{\delta} \quad (18) \\
&= \boldsymbol{x}^\mathsf{T}(\boldsymbol{Z}\boldsymbol{\Sigma}\boldsymbol{Z}^\mathsf{T})^{-1}\boldsymbol{\delta} = \boldsymbol{x}^\mathsf{T}\widetilde{\boldsymbol{\Sigma}}_d^{-1}\boldsymbol{\delta}, \quad (19)
\end{aligned}
$$

where $\boldsymbol{Y} = \boldsymbol{A}\boldsymbol{W} \in \mathbb{R}^{d\times p}$ so that $\boldsymbol{Z} = \boldsymbol{Y}^\mathsf{T}\boldsymbol{Y}$ is a symmetric $p \times p$ matrix of rank d. In other words, rotating and then projecting is equivalent to a change of basis. The implications of the above is:

Lemma 5. *g_A is rotationally invariant if and only if span($\boldsymbol{A}$)=span($\boldsymbol{\Sigma}_d$). In other words, `rrLDA` is the only rotationally invariant projection.*

2.6 Low-Rank Canonical Correlation Analysis

We now contrast LOL and low-rank CCA. For discriminant analysis, low-rank CCA corresponds to finding the eigenvectors of $S_X^{\dagger} S_B$ where

$$S_X = \sum_i (X_i - \bar{X})(X_i - \bar{X})^\top; \quad \bar{X} = \sum_i X_i \tag{20}$$

is the sample covariance matrix of the X_i, $S_X^{\dagger}$ is the inverse of S_X (or Moore-Penrose pseudo-inverse of S_X if S_X is not invertible), and

$$S_B = \frac{n_0}{n}(\bar{X}_0 - \bar{X})(\bar{X}_0 - \bar{X})^\top + \frac{n_1}{n}(\bar{X}_1 - \bar{X})(\bar{X}_1 - \bar{X})^\top; \quad \bar{X}_j = \sum_{i:\, Y_i = j} X_i \text{ for } j \in \{0, 1\} \tag{21}$$

is the between class covariance matrix [18]. It is widely known (see section 11.5 of [16]) that if S_X is invertible then the above formulation reduces to that of Fisher L, namely that of finding $\hat{v}$ satisfying

$$\hat{v} = \operatorname*{argmax}_{v \neq 0} \frac{v^\top S_B v}{v^\top S_W v} \tag{22}$$

$$S_W = \sum_{i:\, Y_i = 0} (X_i - \bar{X}_0)(X_i - \bar{X}_0)^\top + \sum_{i:\, Y_i = 1} (X_i - \bar{X}_1)(X_i - \bar{X}_1)^\top; \tag{23}$$

where S_W is the pooled within-sample covariance matrix and $S_X = S_W + S_B$. In the context of our current paper where X is assumed to be high-dimensional, it is well-known that S_X is not a good estimator of the population covariance matrix $\Sigma_X = \mathbb{E}[(X - \mu)(X - \mu)^\top]$ and thus computing S_X^{-1} is suboptimal for subsequent inference unless some form of regularization is employed. Our consideration of low-rank linear transformations AX provides one principled approach to regularizations of high-dimensional S_X. In contrasts, the above (unregularized) formulation of low-rank CCA frequently yields discrimination direction vectors corresponding to "maximum data piling" (MDP) directions [1, 18] in high-dimensional settings (and always yield maximum data piling directions when $p \geq n$). These MDP directions lead to *perfect* discrimination of the training data, but can suffer from poor generalization performance, as the examples in [1, 18] indicate.

Naïvely computing the low-rank CCA projection requires storing and inverting a $p \times p$ matrix. However, we devised an implementation for low-rank CCA that does not require ever materializing this matrix. Modern eigensolvers computes eigenvalues by performing a sequence of matrix vector multiplication. For example, to compute eigenvalues of S_X, an eigensolver performs $S_X v$ multiple times until the algorithm converges. Assume that the number of iteration is i, the computation complexity of the eigensolver is $O(n \times p \times i)$. Performing pseudo-inverse of S_X computes truncated SVD on S_X, resulting in $S_X v = \sum_i (X_i - \bar{X})((X_i - \bar{X})^\top v)$. Here we never physically generate S_X. Instead, we always compute $v' = (X_i - \bar{X})^\top v$ and then $v'' = (X_i - \bar{X})v'$ to compute $S_X v$. Assume k classes, $S_X v$ has the computation complexity of $O(n \times p \times k)$ and the space complexity of $O(n \times p \times k)$. S_X can be decomposed into $U \Sigma V$, where U is a $n \times n$ matrix and V is a $n \times p$ matrix.

$$S_X^{\dagger} S_B = U \Sigma^{-1} V (\frac{n_0}{n}(\bar{X}_0 - \bar{X})(\bar{X}_0 - \bar{X})^\top + \frac{n_1}{n}(\bar{X}_1 - \bar{X})(\bar{X}_1 - \bar{X})^\top). \tag{24}$$

Computing eigenvalues of $S_X^{\dagger} S_B$ requires

$$S_X^{\dagger} S_B v = U \Sigma^{-1} V (\frac{n_0}{n}(\bar{X}_0 - \bar{X})((\bar{X}_0 - \bar{X})^\top v) + \frac{n_1}{n}(\bar{X}_1 - \bar{X})((\bar{X}_1 - \bar{X})^\top v)). \tag{25}$$

Similar to $S_X v$, we never physically generate $S_X^{\dagger}$ or S_B. Instead, we always multiply the terms on the right with v first, which results in the computation complexity of $O(n \times p)$ and the space complexity of $O(n \times p)$. To our knowledge, this algorithm is novel, and the implementation is also of course novel.

Supplementary Note 3 Simulations

Let $f_{x|y}$ denote the conditional distribution of X given Y, and let f_y denote the prior probability of Y. For simplicity, assume that realizations of the random variable X are p-dimensional vectors, $x \in \mathbb{R}^p$, and realizations of the random variable Y are binary, $y \in \{0, 1\}$. For most simulation settings, each class is Gaussian: $f_{x|y} = \mathcal{N}(\boldsymbol{\mu}_y, \boldsymbol{\Sigma_y})$, where $\boldsymbol{\mu}_y$ is the class-conditional mean and $\boldsymbol{\Sigma}_y$ is the class-conditional covariance. Moreover, we assume f_y is a Bernoulli distribution with probability π that $y = 1$, $f_y = \mathcal{B}(\pi)$. We typically assume that both classes are equally likely, $\pi = 0.5$, and the covariance matrices are the same, $\boldsymbol{\Sigma}_0 = \boldsymbol{\Sigma}_1 = \boldsymbol{\Sigma}$. Under such assumptions, we merely specify $\boldsymbol{\theta} = \{\boldsymbol{\mu}_0, \boldsymbol{\mu}_1, \boldsymbol{\Sigma}\}$. We consider the following simulation settings:

Stacked Cigars

- $\boldsymbol{\mu}_0 = \mathbf{0}$,
- $\boldsymbol{\mu}_1 = (a, b, a, \ldots, a)$,
- $\boldsymbol{\Sigma}$ is a diagonal matrix, with diagonal vector, $\boldsymbol{d} = (1, b, 1 \ldots, 1)$,

where $a = 0.15$ and $b = 4$.

Trunk

- $\boldsymbol{\mu}_0 = b/\sqrt{(1, 3, 5, \ldots, 2p)}$,
- $\boldsymbol{\mu}_1 = -\boldsymbol{\mu}_0$,
- $\boldsymbol{\Sigma}$ is a diagonal matrix, with diagonal vector, $\boldsymbol{d} = 100/\sqrt{(p, p-1, p-2, \ldots, 1)}$,

where $b = 4$.

Rotated Trunk Same as Trunk, but the data are randomly rotated, that is, we sample $\boldsymbol{Q}$ uniformly from the set of p-dimensional rotation matrices, and then set:

- $\boldsymbol{\mu}_0 \leftarrow \boldsymbol{Q}\boldsymbol{\mu}_0$,
- $\boldsymbol{\mu}_1 \leftarrow \boldsymbol{Q}\boldsymbol{\mu}_1$,
- $\boldsymbol{\Sigma} \leftarrow \boldsymbol{Q}\boldsymbol{\Sigma}\boldsymbol{Q}^{\mathsf{T}}$.

3 Classes Same as Trunk, but with a third mean equal to the zero vector, $\boldsymbol{\mu}_2 = \mathbf{0}$.

- $\boldsymbol{\mu}_0 = b/\sqrt{(1, 3, 5, \ldots, 2p)}$,
- $\boldsymbol{\mu}_1 = -\boldsymbol{\mu}_0$,
- $\boldsymbol{\mu}_2 = \mathbf{0}$,
- $\boldsymbol{\Sigma}$ is a diagonal matrix, with diagonal vector, $\boldsymbol{d} = 100/\sqrt{(p, p-1, p-2, \ldots, 1)}$,

where $b = 4$.

Robust An experiment in which outliers are present for estimation of the projection matrix, but removed for training and testing of the classifier. This is due to the strong amount of noise present in the robust experiment will lead to poor generalizability of the estimated `LDA` classifier. Parameters indexed by i correspond to the generative model for the inliers, and those with o correspond to the outliers.

- $\boldsymbol{\mu}_0^{(i)} = b/\sqrt{(1, 3, 5, \ldots, p)}$ for the first $p/2$ dimensions and 0 otherwise,

- $\boldsymbol{\mu}_1^{(i)} = -\boldsymbol{\mu}_0$,
- $\boldsymbol{\Sigma}^{(i)} = b^3/\sqrt{(1, 2, \ldots, p)}$,
- $\boldsymbol{\mu}^{(o)} = 0$,
- $\boldsymbol{\Sigma}^{(o)} = b^6/\sqrt{(1, 2, \ldots, p)}$,
- $\pi^{(i)} = 0.7$,
- $\pi^{(o)} = 0.3$,

and outliers are randomly assigned class 0 or class 1 with equal probability.

Cross An experiment in which the two classes have identical means but different covariance matrices, meaning the optimal discriminant boundary is quadratic.

- $\boldsymbol{\mu}_0 = \boldsymbol{\mu}_1 = \mathbf{0}$,
- Σ_0 is a diagonal matrix, with diagonal $(a, \ldots, a, b, \ldots, b)$ where the first $\frac{d}{3}$ elements are a, and the rest are b,
- Σ_1 is a diagonal matrix, with diagonal $(b, \ldots, b, a, \ldots, a, b, \ldots, b)$ where the middle $\frac{d}{3}$ elements are a, and the others are b,

and we let $a = 1$, and $b = \frac{1}{4}$.

Hump-K An experiment with K classes, in which the class means display an alternating series of humps, and the class covariance is a scalar multiple of the identity.

- $\pi_k = \frac{1}{K}$
- $x_{l,k} = \lfloor -\frac{K}{2} \rfloor$ the left endpoint of the hump
- $x_{r,k} = d - x_{l,K-k+1}$ the right endpoint of the hump
- $x_{m,k} = \left[\frac{x_{l,k} + x_{r,k}}{2} \right]$ the midpoint of the hump
- Let a_k, b_k, c_k be the unique coefficients such that $c_k + b_k x + a_k x^2$ passes through $x_{l,k}$ at $y = 0$, passes through $x_{r,k}$ at $y = b$, and passes through $x_{m,k}$ at $y = 0$.
- Let $\alpha_k = \begin{cases} 1 & k \text{ is odd} \\ -1 & k \text{ is even} \end{cases}$
- for $j = 1, \ldots, d$, let $\mu_{k,j} = \begin{cases} 0 & j \notin [x_{l,k}, x_{r,k}] \\ c_k + b_k j + a_k j^2 & j \in [x_{l,k}, x_{r,k}] \end{cases}$
- $\boldsymbol{\Sigma}$ is a diagonal matrix, with diagonal vector $(\sigma, \ldots, \sigma)$.

where $b = 4$ and $\sigma = \frac{100}{K}$.

Computational Efficiency Experiments These experiments used the Trunk setting, increasing the observed dimensionality.

Hypothesis Testing Experiments We considered two related joint distributions here. The first joint (Diagonal) is described by:

- $\boldsymbol{\mu}_0 = \mathbf{0}$,
- $\widetilde{\boldsymbol{\mu}}_1 \sim \mathcal{N}(\mathbf{0}, \boldsymbol{I})$, $\boldsymbol{\mu}_1 = \widetilde{\boldsymbol{\mu}}_1 / \left\| \widetilde{\boldsymbol{\mu}}_1 \right\|$,
- $\boldsymbol{\Sigma}$ is the same Toeplitz matrix (where the top row is $\rho^{(0,1,2,\ldots,p-1)}$), and the matrix is rescaled to have a Frobenius norm of 50.

The second (Dense) is the same except that the eigenvectors are uniformly random sampled orthonormal matrices, rather than the identity matrix.

Regression Experiments In this experiment we used a distribution similar to the Toeplitz distribution as described above, but y was a linear function of x, that is, $y = Ax$, where $x \sim \mathcal{N}(\mathbf{0}, \Sigma)$, where Σ is the above described Toeplitz matrix, and A is a diagonal matrix whose first two diagonal elements are non-zero, and the rest are zero.

Supplementary Note 4 Theorems and Proofs of Main Result

4.1 Chernoff information

We now introduce the notion of the Chernoff information, which serves as our surrogate measure for the Bayes error of any classification procedure given the *projected* data. Our discussion of the Chernoff information is under the context of decision rules for hypothesis testing, nevertheless, as evidenced by the fact that the *maximum a posteriori* decision rule—equivalently the Bayes classifier—achieves the Chernoff information rate, this distinction between hypothesis testing and classification is mainly for ease of exposition.

Let F_0 and F_1 be two absolutely continuous multivariate distributions in $\Omega \subset \mathbb{R}^d$ with density functions f_0 and f_1, respectively. Suppose that $X_1, X_2, \dots, X_m$ are independent and identically distributed random variables, with X_i distributed either F_0 or F_1. We are interested in testing the simple null hypothesis $\mathbb{H}_0\colon F = F_0$ against the simple alternative hypothesis $\mathbb{H}_1\colon F = F_1$. A test T is a sequence of mapping $T_m\colon \Omega^m \mapsto \{0, 1\}$ such that given $X_1 = x_1, X_2 = x_2, \dots, X_m = x_m$, the test rejects $\mathbb{H}_0$ in favor of $\mathbb{H}_1$ if $T_m(x_1, x_2, \dots, x_m) = 1$; similarly, the test decides $\mathbb{H}_1$ instead of $\mathbb{H}_0$ if $T_m(x_1, x_2, \dots, x_m) = 0$. The Neyman-Pearson lemma states that, given $X_1 = x_1, X_2 = x_2, \dots, X_m = x_m$ and a threshold $\eta_m \in \mathbb{R}$, the likelihood ratio test rejects $\mathbb{H}_0$ in favor of $\mathbb{H}_1$ whenever

$$\Big(\sum_{i=1}^{m} \log f_0(x_i) - \sum_{i=1}^{m} \log f_1(x_i)\Big) \leq \eta_m. \tag{26}$$

Moreover, the likelihood ratio test is the most powerful test at significance level $\alpha_m = \alpha(\eta_m)$, i.e., the likelihood ratio test minimizes the type II error β_m subject to the constraint that the type I error is at most α_m.

Assume that $\pi \in (0, 1)$ is a prior probability of $\mathbb{H}_0$ being true. Then, for a given $\alpha_m^* \in (0, 1)$, let $\beta_m^* = \beta_m^*(\alpha_m^*)$ be the type II error associated with the likelihood ratio test when the type I error is at most α_m^*. The quantity $\inf_{\alpha_m^* \in (0,1)} \pi\alpha_m^* + (1 - \pi)\beta_m^*$ is then the Bayes risk in deciding between $\mathbb{H}_0$ and $\mathbb{H}_1$ given the m independent random variables $X_1, X_2, \dots, X_m$. A classical result of Chernoff [6] states that the Bayes risk is intrinsically linked to a quantity known as the *Chernoff information*. More specifically, let $C(F_0, F_1)$ be the quantity

$$\begin{aligned} C(F_0, F_1) &= -\log \Big[\inf_{t \in (0,1)} \int_{\mathbb{R}^d} f_0^t(\boldsymbol{x}) f_1^{1-t}(\boldsymbol{x}) \mathrm{d}\boldsymbol{x}\Big] \\ &= \sup_{t \in (0,1)} \Big[-\log \int_{\mathbb{R}^d} f_0^t(\boldsymbol{x}) f_1^{1-t}(\boldsymbol{x}) \mathrm{d}\boldsymbol{x}\Big] \end{aligned} \tag{27}$$

Then we have

$$\lim_{m \to \infty} \frac{1}{m} \inf_{\alpha_m^* \in (0,1)} \log(\pi\alpha_m^* + (1 - \pi)\beta_m^*) = -C(F_0, F_1). \tag{28}$$

Thus $C(F_0, F_1)$ is the *exponential* rate at which the Bayes error $\inf_{\alpha_m^* \in (0,1)} \pi\alpha_m^* + (1-\pi)\beta_m^*$ decreases as $m \to \infty$; we also note that the $C(F_0, F_1)$ is independent of π. We also define, for a given $t \in (0,1)$ the Chernoff divergence $C_t(F_0, F_1)$ between F_0 and F_1 by

$$C_t(F_0, F_1) = -\log \int_{\mathbb{R}^d} f_0^t(\boldsymbol{x}) f_1^{1-t}(\boldsymbol{x}) \mathrm{d}\boldsymbol{x}. \tag{29}$$

The Chernoff divergence is an example of a f-divergence as defined in [8]. When $t = 1/2$, $C_t(F_0, F_1)$ is the Bhattacharyya distance between F_0 and F_1.

The result of Supp. Eq. (28) can be extended to $K + 1 \geq 2$ hypothesis, with the exponential rate being the minimum of the Chernoff information between any pair of hypothesis. More specifically, let $F_0, F_1, \dots, F_K$ be distributions on $\mathbb{R}^d$ and let $X_1, X_2, \dots, X_m$ be independent and identically distributed random variables with distribution $F \in \{F_0, F_1, \dots, F_K\}$. Our inference task is in determining the distribution of the X_i among the $K+1$ hypothesis $\mathbb{H}_0 \colon F = F_0, \dots, \mathbb{H}_K \colon F = F_K$. Suppose also that hypothesis $\mathbb{H}_k$ has *a priori* probability π_k. For any decision rule g, the risk of g is $r(g) = \sum_k \pi_k \sum_{l \neq k} \alpha_{lk}(g)$ where $\alpha_{lk}(g)$ is the probability of accepting hypothesis $\mathbb{H}_l$ when hypothesis $\mathbb{H}_k$ is true. Then we have [13]

$$\inf_{g} \lim_{m \to \infty} \frac{r(g)}{m} = -\min_{k \neq l} C(F_k, F_l), \tag{30}$$

where the infimum is over all decision rules g, i.e., for any g, $r(g)$ decreases to 0 as $m \to \infty$ at a rate no faster than $\exp(-m \min_{k \neq l} C(F_k, F_l))$.

When the distributions F_0 and F_1 are multivariate normal, that is, $F_0 = \mathcal{N}(\mu_0, \Sigma_0)$ and $F_1 = \mathcal{N}(\mu_1, \Sigma_1)$; then, denoting by $\Sigma_t = t\Sigma_0 + (1-t)\Sigma_1$, we have

$$C(F_0, F_1) = \sup_{t \in (0,1)} \Big(\frac{t(1-t)}{2} (\mu_1 - \mu_2)^\top \Sigma_t^{-1} (\mu_1 - \mu_2) + \frac{1}{2} \log \frac{|\Sigma_t|}{|\Sigma_0|^t |\Sigma_1|^{1-t}} \Big). \tag{31}$$

4.2 Projecting data and Chernoff information

We now discuss how the Chernoff information characterizes the effect a linear transformation A of the data has on classification accuracy. We start with the following simple result whose proof follows directly from Supp. Eq. (30).

Lemma 6. *Let $F_0 = \mathcal{N}(\mu_0, \Sigma)$ and $F_1 \sim \mathcal{N}(\mu_1, \Sigma)$ be two multivariate normals with equal covariance matrices. For any linear transformation A, let $F_0^{(A)}$ and $F_1^{(A)}$ denote the distribution of AX when $X \sim F_0$ and $X \sim F_1$, respectively. We then have*

$$\begin{aligned} C(F_0^{(A)}, F_1^{(A)}) &= \frac{1}{8} (\mu_1 - \mu_0)^\top A^\top (A \Sigma A^\top)^{-1} A (\mu_1 - \mu_0) \\ &= \frac{1}{8} (\mu_1 - \mu_0)^\top \Sigma^{-1/2} \Sigma^{1/2} A^\top (A \Sigma A^\top)^{-1} A \Sigma^{1/2} \Sigma^{-1/2} (\mu_1 - \mu_0) \\ &= \frac{1}{8} \| P_{\Sigma^{1/2} A^\top} \Sigma^{-1/2} (\mu_1 - \mu_0) \|_F^2 \end{aligned} \tag{32}$$

where $P_Z = Z(Z^\top Z)^{-1} Z^\top$ denotes the matrix corresponding to the orthogonal projection onto the columns of Z.

Thus for a classification problem where $X|Y = 0$ and $X|Y = 1$ are distributed multivariate normals with mean μ_0 and μ_1 and the same covariance matrix Σ, Lemma 6 then states that for any two linear

transformations A and B, the transformed data AX is to be preferred over the transformed data BX if

$$(\mu_1 - \mu_0)^\top A^\top (A\Sigma A^\top)^{-1} A(\mu_1 - \mu_0) > (\mu_1 - \mu_0)^\top B^\top (B\Sigma B^\top)^{-1} B(\mu_1 - \mu_0). \tag{33}$$

In particular, using Lemma 6, we obtain the following result showing the dominance of `LOL` over reduced-rank `LDA` (or simply `rrLDA` for brevity) when the class conditional distributions are multivariate normal with a common variance.

Theorem 1. *Let $F_0 = N(\mu_0, \Sigma)$ and $F_1 \sim N(\mu_1, \Sigma)$ be multivariate normal distributions in $\mathbb{R}^p$. Let $\lambda_1 \geq \lambda_2 \geq \dots \lambda_p$ be the eigenvalues of Σ and $u_1, u_2, \dots, u_p$ the corresponding eigenvectors. For $d \leq p$, let $U_d = [u_1 \mid u_2 \mid \cdots \mid u_d] \in \mathbb{R}^{p \times d}$ be the matrix whose columns are the eigenvectors $u_1, u_2, \dots, u_d$. Let $A = [\delta \mid U_{d-1}]$ and $B = U_d$ be the `LOL` and `rrLDA` linear transformations into $\mathbb{R}^d$, respectively. Then*

$$\begin{aligned} C(F_0^{(A)}, F_1^{(A)}) - C(F_0^{(B)}, F_1^{(B)}) &= \frac{(\delta^\top (I - U_{d-1}U_{d-1}^\top)\delta)^2}{\delta^\top(\Sigma - \Sigma_{d-1})\delta} - \delta^\top(\Sigma_d^\dagger - \Sigma_{d-1}^\dagger)\delta \\ &\geq \frac{1}{\lambda_d}\delta^\top(I - U_{d-1}U_{d-1}^\top)\delta - \frac{1}{\lambda_d}\delta^\top(U_dU_d^\top - U_{d-1}U_{d-1}^\top)\delta \geq 0 \end{aligned} \tag{34}$$

and the inequality is strict whenever $\delta^\top(I - U_dU_d^\top)\delta > 0$.

Proof. We first note that

$$A\Sigma A^\top = \left[\delta | U_{d-1}\right]^\top \Sigma \left[\delta | U_{d-1}\right] = \begin{bmatrix} \delta^\top \Sigma \delta & \delta^\top \Sigma U_{d-1} \\ U_{d-1}^\top \Sigma \delta & U_{d-1}^\top \Sigma U_{d-1} \end{bmatrix} = \begin{bmatrix} \delta^\top \Sigma \delta & \delta^\top \Sigma U_{d-1} \\ U_{d-1}^\top \Sigma \delta & \Lambda_{d-1} \end{bmatrix} \tag{35}$$

where $\Lambda_{d-1} = \mathrm{diag}(\lambda_1, \lambda_2, \dots, \lambda_{d-1})$ is the $(d-1) \times (d-1)$ diagonal matrix formed by the eigenvalues $\lambda_1, \lambda_2, \dots, \lambda_{d-1}$. Therefore, letting $\gamma = \delta^\top\Sigma\delta - \delta^\top\Sigma U_{d-1}\Lambda_{d-1}^{-1}U_{d-1}^\top\Sigma\delta$, we have

$$\begin{aligned} (A\Sigma A^\top)^{-1} &= \begin{bmatrix} \delta^\top \Sigma \delta & \delta^\top \Sigma U_{d-1} \\ U_{d-1}^\top \Sigma \delta & U_{d-1}^\top \Sigma U_{d-1} \end{bmatrix}^{-1} \\ &= \begin{bmatrix} \gamma^{-1} & -\delta^\top \Sigma U_{d-1} \Lambda_{d-1}^{-1} \gamma^{-1} \\ -\Lambda_{d-1}^{-1} U_{d-1}^\top \Sigma \delta \gamma^{-1} & \left(\Lambda_{d-1} - \frac{U_{d-1}^\top \Sigma\delta\delta^\top\Sigma U_{d-1}}{\delta^\top\Sigma\delta}\right)^{-1} \end{bmatrix}. \end{aligned} \tag{36}$$

The Sherman-Morrison-Woodbury formula then implies

$$\begin{aligned} \left(\Lambda_{d-1} - \frac{U_{d-1}^\top\Sigma\delta\delta^\top\Sigma U_{d-1}}{\delta^\top\Sigma\delta}\right)^{-1} &= \Lambda_{d-1}^{-1} + \frac{\Lambda_{d-1}^{-1}U_{d-1}^\top\Sigma\delta\delta^\top\Sigma U_{d-1}\Lambda_{d-1}^{-1}/(\delta^\top\Sigma\delta)}{1 - \delta^\top\Sigma U_{d-1}\Lambda_{d-1}^{-1}U_{d-1}^\top\Sigma\delta/(\delta^\top\Sigma\delta)} \\ &= \Lambda_{d-1}^{-1} + \frac{\Lambda_{d-1}^{-1}U_{d-1}^\top\Sigma\delta\delta^\top\Sigma U_{d-1}\Lambda_{d-1}^{-1}}{\delta^\top\Sigma\delta - \delta^\top\Sigma U_{d-1}\Lambda_{d-1}^{-1}U_{d-1}^\top\Sigma\delta} \\ &= \Lambda_{d-1}^{-1} + \gamma^{-1}\Lambda_{d-1}^{-1}U_{d-1}^\top\Sigma\delta\delta^\top\Sigma U_{d-1}\Lambda_{d-1}^{-1} \end{aligned} \tag{37}$$

We note that $\Sigma U_{d-1} = U_{d-1}\Lambda_{d-1}$ and $U_{d-1}^\top\Sigma = \Lambda_{d-1}U_{d-1}^\top$ and hence

$$\begin{aligned} \gamma = \delta^\top\Sigma\delta - \delta^\top\Sigma U_{d-1}\Lambda_{d-1}^{-1}U_{d-1}^\top\Sigma\delta &= \delta^\top\Sigma\delta - \delta^\top U_{d-1}\Lambda_{d-1}\Lambda_{d-1}^{-1}\Lambda_{d-1}U_{d-1}^\top\delta \\ &= \delta^\top\Sigma\delta - \delta^\top U_{d-1}\Lambda_{d-1}U_{d-1}^\top\delta = \delta^\top(\Sigma - \Sigma_{d-1})\delta \end{aligned} \tag{38}$$

where $\Sigma_{d-1} = U_{d-1}\Lambda_{d-1}U_{d-1}^\top$ is the best rank $d-1$ approximation to Σ with respect to any unitarily invariant norm. In addition,

$$\Lambda_{d-1}^{-1}U_{d-1}^\top \Sigma\delta\delta^\top\Sigma U_{d-1}\Lambda_{d-1}^{-1} = \Lambda_{d-1}^{-1}\Lambda_{d-1}U_{d-1}^\top\delta\delta^\top U_{d-1}\Lambda_{d-1}\Lambda_{d-1}^{-1} = U_{d-1}^\top\delta\delta^\top U_{d-1}.$$

We thus have

$$(A\Sigma A^\top)^{-1} = \begin{bmatrix} \gamma^{-1} & -\delta^\top\Sigma U_{d-1}\Lambda_{d-1}^{-1}\gamma^{-1} \\ -\Lambda_{d-1}^{-1}U_{d-1}^\top\Sigma\delta\gamma^{-1} & \big(\Lambda_{d-1} - \frac{U_{d-1}^\top\Sigma\delta\delta^\top\Sigma U_{d-1}}{\delta^\top\Sigma\delta}\big)^{-1} \end{bmatrix} \tag{39}$$

$$= \begin{bmatrix} \gamma^{-1} & -\gamma^{-1}\delta^\top U_{d-1} \\ -\gamma^{-1}U_{d-1}^\top\delta & \Lambda_{d-1}^{-1} + \gamma^{-1}U_{d-1}^\top\delta\delta^\top U_{d-1} \end{bmatrix}. \tag{40}$$

Therefore,

$$\begin{aligned} \delta^\top A^\top(A\Sigma A^\top)^{-1}A\delta &= \delta^\top\big[\delta \mid U_{d-1}\big] \begin{bmatrix} \gamma^{-1} & -\gamma^{-1}\delta^\top U_{d-1} \\ -\gamma^{-1}U_{d-1}^\top\delta & \Lambda_{d-1}^{-1} + \gamma^{-1}U_{d-1}^\top\delta\delta^\top U_{d-1} \end{bmatrix} \big[\delta|U_{d-1}\big]^\top\delta \\ &= \big[\delta^\top\delta \mid \delta^\top U_{d-1}\big] \begin{bmatrix} \gamma^{-1} & -\gamma^{-1}\delta^\top U_{d-1} \\ -\gamma^{-1}U_{d-1}^\top\delta & \Lambda_{d-1}^{-1} + \gamma^{-1}U_{d-1}^\top\delta\delta^\top U_{d-1} \end{bmatrix} \begin{bmatrix} \delta^\top\delta \\ U_{d-1}^\top\delta \end{bmatrix} \\ &= \gamma^{-1}(\delta^\top\delta)^2 - 2\gamma^{-1}\delta^\top\delta\delta^\top U_{d-1}U_{d-1}^\top\delta + \delta^\top U_{d-1}(\Lambda_{d-1}^{-1} + \gamma^{-1}U_{d-1}^\top\delta\delta^\top U_{d-1})U_{d-1}^\top\delta \\ &= \gamma^{-1}(\delta^\top\delta - \delta^\top U_{d-1}U_{d-1}^\top\delta)^2 + \delta^\top U_{d-1}\Lambda_{d-1}^{-1}U_{d-1}^\top\delta \\ &= \gamma^{-1}(\delta^\top(I - U_{d-1}U_{d-1}^\top)\delta)^2 + \delta^\top\Sigma_{d-1}^\dagger\delta \end{aligned} \tag{41}$$

where $\Sigma_{d-1}^\dagger$ is the Moore-Penrose pseudo-inverse of Σ_{d-1}. The $\texttt{LDA}$ projection matrix into $\mathbb{R}^d$ is given by $B = U_d^\top$ and hence

$$\delta^\top B^\top(B\Sigma B^\top)^{-1}B\delta = \delta^\top U_d\Lambda_d^{-1}U_d^\top\delta = \delta^\top\Sigma_d^\dagger\delta. \tag{42}$$

We thus have

$$\begin{aligned} C(F_0^{(A)}, F_1^{(A)}) - C(F_0^{(B)}, F_1^{(B)}) &= \gamma^{-1}(\delta^\top(I - U_{d-1}U_{d-1}^\top)\delta)^2 - \delta^\top(\Sigma_d^\dagger - \Sigma_{d-1}^\dagger)\delta \\ &= \frac{(\delta^\top(I - U_{d-1}U_{d-1}^\top)\delta)^2}{\delta^\top(\Sigma - \Sigma_{d-1})\delta} - \delta^\top(\Sigma_d^\dagger - \Sigma_{d-1}^\dagger)\delta \\ &\geq \frac{(\delta^\top(I - U_{d-1}U_{d-1}^\top)\delta)^2}{\lambda_d\delta^\top(I - U_{d-1}U_{d-1}^\top)\delta} - \frac{1}{\lambda_d}\delta^\top u_d u_d^\top\delta \\ &= \frac{1}{\lambda_d}\delta^\top(I - U_{d-1}U_{d-1})\delta - \frac{1}{\lambda_d}\delta^\top(U_dU_d^\top - U_{d-1}U_{d-1}^\top)\delta \geq 0 \end{aligned} \tag{43}$$

where we recall that u_d is the d-th column of U_d. Thus $C(F_0^{(A)}, F_1^{(A)}) \geq C(F_0^{(B)}, F_1^{(B)})$ always, and the inequality is strict whenever $\delta^\top(I - U_dU_d^\top)\delta > 0$. □

Remark 1. *Theorem 1 can be extended to the case wherein the linear transformations are $A = [\delta \mid U_{d-1}]$ and $B = U_d$, respectively, such that U_d is an arbitrary $p \times d$ matrix with $U_d^\top U_d = I$, and U_{d-1} is the first $d-1$ columns of U_d. A similar derivation to that in the proof of Theorem 1 then yields*

$$C(F_0^{(A)}, F_1^{(A)}) = \frac{(\delta^\top\Sigma^{-1/2}(I - V_{d-1}V_{d-1}^\top)\Sigma^{1/2}\delta)^2}{\delta^\top\Sigma^{1/2}(I - V_{d-1}V_{d-1}^\top)\Sigma^{1/2}\delta} + \delta^\top\Sigma^{-1/2}V_{d-1}V_{d-1}^\top\Sigma^{-1/2}\delta$$

$$C(F_0^{(B)}, F_1^{(B)}) = \delta^\top\Sigma^{-1/2}V_dV_d^\top\Sigma^{-1/2}\delta$$

where $V_d V_{d-1}^\top = \Sigma^{1/2} U_d (U_d^\top \Sigma U_d)^{-1} U_d^\top \Sigma^{1/2}$ is the orthogonal projection onto the column space of $\Sigma^{1/2} U_d$. Hence $C(F_0^{(A)}, F_1^{(A)}) > C(F_0^{(B)}, F_1^{(B)})$ if and only if

$$\frac{(\delta^\top \Sigma^{-1/2} (I - V_{d-1} V_{d-1}^\top) \Sigma^{1/2} \delta)^2}{\delta^\top \Sigma^{1/2} (I - V_{d-1} V_{d-1}^\top) \Sigma^{1/2} \delta} > \delta^\top \Sigma^{-1/2} (V_d V_d^\top - V_{d-1} V_{d-1}^\top) \Sigma^{-1/2} \delta. \tag{44}$$

We recover Supp. Eq. 34 by letting U_d be the matrix whose columns are the eigenvectors corresponding to the d largest eigenvalue of Σ.

We next present a result relating the Chernoff information for `LOL` and `rrLDA`.

Theorem 2. *Assume the setting of Theorem 1. Let $C = \tilde{U}_d^\top$ where $\tilde{U}_d$ is the $p \times d$ matrix whose columns are the d largest eigenvectors of the* pooled *covariance matrix $\tilde{\Sigma} = \mathbb{E}[(X - \frac{\mu_0 + \mu_1}{2})(X - \frac{\mu_0 + \mu_1}{2})^\top]$. Then C is the linear transformation for `PCA` and*

$$\begin{aligned} C(F_0^{(A)}, F_1^{(A)}) - C(F_0^{(C)}, F_1^{(C)}) &= \frac{(\delta^\top (I - U_{d-1} U_{d-1}^\top) \delta)^2}{\delta^\top (\Sigma - \Sigma_{d-1}) \delta} + \delta^\top \Sigma_{d-1}^\dagger \delta - \delta^\top \tilde{\Sigma}_d^\dagger \delta - \frac{(\delta^\top \tilde{\Sigma}_d^\dagger \delta)^2}{4 - \delta^\top \tilde{\Sigma}_d^\dagger \delta} \\ &= \frac{(\delta^\top (I - U_{d-1} U_{d-1}^\top) \delta)^2}{\delta^\top (\Sigma - \Sigma_{d-1}) \delta} + \delta^\top \Sigma_{d-1}^\dagger \delta - \frac{4 \delta^\top \tilde{\Sigma}_d^\dagger \delta}{4 - \delta^\top \tilde{\Sigma}_d^\dagger \delta}. \end{aligned} \tag{45}$$

where $\tilde{\Sigma}_d = \tilde{U}_d \tilde{S}_d \tilde{U}_d^\top$ is the best rank d approximation to $\tilde{\Sigma} = \Sigma + \frac{1}{4} \delta \delta^\top$.

Proof. Assume, without loss of generality, that $\mu_1 = -\mu_0 = \mu$. We then have

$$\tilde{\Sigma} = \mathbb{E}[X X^\top] = \pi \Sigma + \pi \mu_0 \mu_0^\top + (1 - \pi) \Sigma + (1 - \pi) \mu_1 \mu_1^\top = \Sigma + \mu \mu^\top = \Sigma + \tfrac{1}{4} \delta \delta^\top. \tag{46}$$

Therefore

$$(C \Sigma C^\top)^{-1} = \big(\tilde{U}_d^\top \Sigma \tilde{U}_d\big)^{-1} = \big(\tilde{U}_d^\top (\tilde{\Sigma} - \tfrac{1}{4} \delta \delta^\top) \tilde{U}_d\big)^{-1} = \big(\tilde{S}_d - \tfrac{1}{4} \tilde{U}_d^\top \delta \delta^\top \tilde{U}_d\big)^{-1} \tag{47}$$

$$= \tilde{S}_d^{-1} + \frac{\tilde{S}_d^{-1} \tilde{U}_d^\top \delta \delta^\top \tilde{U}_d \tilde{S}_d^{-1}}{4 - \delta^\top \tilde{U}_d \tilde{S}_d^{-1} \tilde{U}_d^\top \delta} \tag{48}$$

where $\tilde{S}_d$ is the diagonal matrix containing the d largest eigenvalues of $\tilde{\Sigma}$. Hence

$$\begin{aligned} C(F_0^{(C)}, F_1^{(C)}) = \delta^\top C^\top (C \Sigma C^\top)^{-1} C \delta &= \delta^\top \tilde{U}_d \Big(\tilde{S}_d^{-1} + \frac{\tilde{S}_d^{-1} \tilde{U}_d^\top \delta \delta^\top \tilde{U}_d \tilde{S}_d^{-1}}{4 - \delta^\top \tilde{U}_d \tilde{S}_d^{-1} \tilde{U}_d^\top \delta}\Big) \tilde{U}_d^\top \delta \\ &= \delta^\top \tilde{U}_d \tilde{S}_d^{-1} \tilde{U}_d^\top \delta + \frac{(\delta^\top \tilde{U}_d \tilde{S}_d^{-1} \tilde{U}_d^\top \delta)^2}{4 - \delta^\top \tilde{U}_d \tilde{S}_d^{-1} \tilde{U}_d^\top \delta} \\ &= \delta^\top \tilde{\Sigma}_d^\dagger \delta + \frac{(\delta^\top \tilde{\Sigma}_d^\dagger \delta)^2}{4 - \delta^\top \tilde{\Sigma}_d^\dagger \delta} = \frac{4 \delta^\top \tilde{\Sigma}_d^\dagger \delta}{4 - \delta^\top \tilde{\Sigma}_d^\dagger \delta}. \end{aligned} \tag{49}$$

as desired. □

Remark 2. *We recall that the `LOL` projection $A = [\delta \mid U_{d-1}]^\top$ yields*

$$C(F_0^{(A)}, F_1^{(A)}) = \frac{(\delta^\top (I - U_{d-1} U_{d-1}^\top) \delta)^2}{\delta^\top (\Sigma - \Sigma_{d-1}) \delta} + \delta^\top \Sigma_{d-1}^\dagger \delta. \tag{50}$$

To illustrate the difference between the `LOL` projection and that based on the eigenvectors of the pooled *covariance matrix, consider the following simple example. Let* $\Sigma = \mathrm{diag}(\lambda_1, \lambda_2, \dots, \lambda_p)$ *be a diagonal matrix with* $\lambda_1 \geq \lambda_2 \geq \cdots \geq \lambda_p$*. Also let* $\delta = (0, 0, \dots, 0, s)$*. Suppose furthermore that* $\lambda_p + s^2/4 < \lambda_d$*. Then we have* $\tilde{\Sigma}_d = \mathrm{diag}(\lambda_1, \lambda_2, \dots, \lambda_d, 0, 0, \dots, 0)$*. Thus* $\tilde{\Sigma}_d^{\dagger} = \mathrm{diag}(1/\lambda_1, 1/\lambda_2, \dots, 1/\lambda_d, 0, 0, \dots, 0)$ *and* $\delta^{\dagger}\tilde{\Sigma}_d^{\dagger}\delta = 0$*. Therefore,* $C(F_0^{(B)}, F_1^{(B)}) = 0$*.*

On the other hand, we have

$$C(F_0^{(A)}, F_1^{(A)}) = \frac{(\delta^{\top}(I - U_{d-1}U_{d-1}^{\top})\delta)^2}{\delta^{\top}(\Sigma - \Sigma_{d-1})\delta} + \delta^{\top}\Sigma_{d-1}^{\dagger}\delta = \frac{s^4}{s^2\lambda_p} + 0 = s^2/\lambda_p. \tag{51}$$

A more general form of the previous observation is the following result which shows that `LOL` is preferable over `PCA` when the dimension p is sufficiently large.

Proposition 1. *Let* Σ *be a* $p \times p$ *covariance matrix of the form*

$$\Sigma = \begin{bmatrix} \Sigma_d & 0 \\ 0 & \Sigma_d^{\perp} \end{bmatrix} \tag{52}$$

where Σ_d *is a* $d \times d$ *matrix. Let* $\lambda_1 \geq \lambda_2 \geq \cdots \geq \lambda_p$ *be the eigenvalues of* Σ*, with* $\lambda_1, \lambda_2, \dots, \lambda_d$ *being the eigenvalues of* Σ_d*. Suppose that the entries of* δ *are i.i.d. with the following properties.*

1. $\delta_i \sim Y_i * N(\tau, \sigma^2)$ *where* $Y_1, Y_2, \dots, Y_p \overset{\text{i.i.d.}}{\sim} \mathrm{Bernoulli}(1-\epsilon)$*.*
2. $p(1-\epsilon) \to \theta$ *as* $p \to \infty$ *for some constant* θ*.*

Then there exists a constant $C > 0$ *such that if* $\lambda_d - \lambda_{d+1} \geq C\theta\tau^2 \log p$*, then, with probability at least* ϵ^d

$$C(F_0^{(A)}, F_1^{(A)}) > C(F_0^{(B)}, F_1^{(B)}) = 0 \tag{53}$$

Proof. The above construction of Σ and δ implies, with probability at least ϵ^d, that the covariance matrix for $\tilde{\Sigma}$ is of the form

$$\tilde{\Sigma} = \begin{bmatrix} \Sigma_d & 0 \\ 0 & \Sigma_d^{\perp} + \frac{1}{4}(\tilde{\delta}\tilde{\delta}^{\top}) \end{bmatrix} \tag{54}$$

where $\tilde{\delta} \in \mathbb{R}^{p-d}$ is formed by excluding the first d elements of δ. Now, if $\lambda_{d+1} + \frac{1}{4}\|\tilde{\delta}\|^2 < \lambda_d$, then the d largest eigenvalues of $\tilde{\Sigma}$ are still $\lambda_1, \lambda_2, \dots, \lambda_d$, and thus the eigenvectors corresponding to the d largest eigenvalues of $\tilde{\Sigma}$ are the same as those for the d largest eigenvalues of Σ. That is to say,

$$\lambda_{d+1} + \tfrac{1}{4}\|\tilde{\delta}\|^2 < \lambda_d \Longrightarrow \tilde{\Sigma}_d^{\dagger} = \Sigma_d^{\dagger} \Longrightarrow \delta^{\top}\tilde{\Sigma}_d^{\dagger}\delta = 0 \Longrightarrow C(F_0^{(B)}, F_1^{(B)}) = 0. \tag{55}$$

We now compute the probability that $\lambda_{d+1} + \frac{1}{4}\|\tilde{\delta}\|^2 < \lambda_d$. Suppose for now that $\epsilon > 0$ is fixed and does not vary with p. We then have

$$\frac{\sum_{i=d+1}^{p} \delta_i^2 - (p-d)(1-\epsilon)\tau^2}{\sqrt{(p-d)(2(1-\epsilon)(2\tau^2\sigma^2 + \sigma^4) + \epsilon(1-\epsilon)(\tau^4 + 2\tau^2\sigma^2 + \sigma^4))}} \xrightarrow{\text{d}} N(0,1). \tag{56}$$

Thus, as $p \to \infty$, the probability that $\lambda_{d+1} + \frac{1}{4}\|\tilde{\delta}\|^2 < \lambda_d$ converges to that of

$$\Phi\Big(\frac{4(\lambda_d - \lambda_{d+1}) - (p-d)(1-\epsilon)\tau^2}{\sqrt{(p-d)(2(1-\epsilon)(2\tau^2\sigma^2 + \sigma^4) + \epsilon(1-\epsilon)(\tau^4 + 2\tau^2\sigma^2 + \sigma^4))}}\Big). \tag{57}$$

This probability can be made arbitrarily close to 1 provided that $\lambda_d - \lambda_{d+1} \geq Cp(1-\epsilon)\tau^2$ for all sufficiently large p and for some constant $C > 1/4$. Since the probability that $\delta_1 = \delta_2 = \cdots = \delta_d$ is at least ϵ^d, we thus conclude that for sufficiently large p, with probability at least ϵ^d,

$$C(F_0^{(B)}, F_1^{(B)}) = 0 < C(F_0^{(A)}, F_1^{(A)}). \tag{58}$$

In the case where $\epsilon = \epsilon(p) \to 1$ as $p \to \infty$ such that $p(1-\epsilon) \to \theta$ for some constant θ, then the probability that $\lambda_{d+1} + \frac{1}{4}\|\tilde{\delta}\|^2 < \lambda_d$ converges to the probability that

$$\frac{1}{4}\sum_{i=1}^{K} \sigma^2 \chi_1^2(\tau) \geq \lambda_d - \lambda_{d+1} \tag{59}$$

where K is Poisson distributed with mean θ and $\chi_i^2(\tau)$ is the non-central chi-square distribution with one degree of freedom and non-centrality parameter τ. Thus if $\lambda_d - \lambda_{d+1} \geq C\theta\tau^2 \log p$ for sufficiently large p and for some constant C, then this probability can also be made arbitrarily close to 1. □

Remark 3. *The previous comparisons are done for the case of $C = 2$ classes. Extending these comparisons to the case of $C > 2$ classes is, however, non-trivial. More precisely, suppose we have $Y \in \{1, 2, \dots, C\}$ and that, conditional on $Y = c$, $X \sim \mathcal{N}(\mu_c, \Sigma)$ is multivariate normal with mean μ_c and* common *covariance matrix Σ. Then, given $X = x$, the Bayes optimal classifier for Y is still*

$$g_{\mathrm{LDA}}(x) = \underset{y \in \{1,2,\dots,C\}}{\operatorname{argmin}} \Big[\frac{1}{2}(x-\mu_y)^\top \Sigma^{-1}(x-\mu_y) - \log \pi_y\Big] \tag{60}$$

$$= \underset{y \in \{1,2,\dots,C\}}{\operatorname{argmin}} \Big[-x^\top \Sigma^{-1}\mu_y + \frac{1}{2}\mu_y^\top \Sigma^{-1}\mu_y - \log \pi_y\Big] \tag{61}$$

Taking $\frac{1}{2}\mu_y^\top \Sigma^{-1}\mu_y - \log \pi_y$ as either a given constant or as an intercept term to be learned or estimated, the reduced-rank LDA for $C > 2$ classes still corresponds to looking at the top d eigenvectors of Σ. That is to say, we transform the predictor variables via $x \mapsto U_d x$ followed by performing LDA on the transformed data. Similarly, the PCA transformation corresponds to using the top d eigenvectors of the pooled covariance matrix $\tilde{\Sigma} = \mathbb{E}[(X - \sum_c \pi_c\mu_c)(X - \sum_c \pi_c\mu_c)^\top]$ followed by performing LDA. Suppose we now compare LOL, rrLDA, and PCA in this multi-class setting. Let $A \colon X \mapsto AX$ be a linear transformation. Then by Supp. Eq. (30) and Supp. Eq. (32), the Chernoff information for the transformed data in this multi-class setting is

$$\min_{c \neq c'} \frac{1}{8}(\mu_c - \mu_{c'})^\top A^\top (A\Sigma A^\top)^{-1} A(\mu_c - \mu_{c'}). \tag{62}$$

We now see that, in the case of rrLDA and PCA the linear transformation A depends only on the covariance matrix Σ and $\tilde{\Sigma}$, respectively. That is to say, the linear transformation A does not depend on the choice of c and c'. In contrast, currently for LOL the linear transformation A depends on both Σ as well as $\mu_c - \mu_{c'}$. In other words, there is no single choice for A but rather that A changes as c, c' changes. Direct comparison, in the multi-classes setting, between LOL and either of rrLDA or PCA is thus an open problem that we leave for future work. Finally we note that if we allow the linear transformation for LOL to vary with the classes c and c', i.e., taking a one-vs-one approach to multi-classes classification, then the resulted presented in this paper are valid for all pairs c, c'.

4.3 Finite Sample Performance

We now consider the finite sample performance of LOL and PCA-based classifiers in the high-dimensional setting with small or moderate sample sizes, e.g., when p is comparable to n or when $n \ll p$. Once again we assume that $X|Y = i \sim \mathcal{N}(\mu_i, \Sigma)$ for $i = 0, 1$. Furthermore, we also assume that Σ belongs to the class $\Theta(p, r, k, \tau, \lambda)$ as defined below.

Definition Let $\lambda > 0, \tau \geq 1$ and $k \leq p$ be given. Denote by $\Theta(p, r, k, \tau, \lambda, \sigma^2)$ the collection of matrices Σ such that

$$\Sigma = V \Lambda V^\top + \sigma^2 I \tag{63}$$

where V is a $p \times r$ matrix with orthonormal columns and Λ is a $r \times r$ diagonal matrix whose diagonal entries $\lambda_1, \lambda_2, \dots, \lambda_r$ satisfy $\lambda \geq \lambda_1 \geq \lambda_2 \geq \cdots \geq \lambda_r \geq \lambda/\tau$. In addition, assume also that $|\mathrm{supp}(V)| \leq k$ where $\mathrm{supp}(V)$ denote the non-zero rows of V, i.e., $\mathrm{supp}(V)$ is the subset of $\{1, 2, \dots, p\}$ such that $V_j \neq 0$ if and only if $j \in \mathrm{supp}(V)$.

We note that in general $r \leq k \ll p$ and $\lambda/\tau \gg \sigma^2$. We then have the following result.

Theorem 3 ([4]). *Suppose there exist constants M_0 and M_1 such that $M_1 \log p \geq \log n \geq M_0 \log \lambda$. Then there exists a constant $c_0 = c_0(M_0, M_1)$ depending on M_0 and M_1 such that for all n and p for which*

$$\frac{\tau k}{n} \log \frac{ep}{k} \leq c_0, \tag{64}$$

there exists an estimate $\hat{V}$ of V such that

$$\sup_{\Sigma \in \Theta(p, r, k, \tau, \lambda, \sigma^2)} \mathbb{E} \|\hat{V}\hat{V}^\top - VV^\top\|^2 \leq \frac{Ck(\sigma\lambda + \sigma^2)}{n\lambda^2} \log \frac{ep}{k} \tag{65}$$

where C is a universal constant not depending on p, r, k, τ, λ and σ^2.

Theorem 3 then implies the following result for comparing the Chernoff information of the sample version of LOL against that for PCA.

Corollary 4. *Let $\Sigma \in \Theta(p, r, k, \tau, \lambda)$ as defined above. Suppose that $C(F_0^{(A)}, F_1^{(A)}) > C(F_0^{(B)}, F_1^{(B)})$ where A and B denote the LOL and PCA projection matrices based on the eigenvectors of Σ associated with the $d \leq r$ largest eigenvalues, i.e, $A = [\delta | V_{1:d-1}]$ and $B = V_{1:d}$. Then there exists constants M and c such that if $\log n \geq M \log \lambda$ and $\frac{\tau k}{n} \log \frac{ep}{k} \leq c$, then there exists an estimate $\hat{V}$ of V such that, with $\hat{A} = [\hat{\delta} | \hat{V}_{1:d-1}]$ and $\hat{B} = [\hat{V}_{1:d}]$, we have*

$$\mathbb{E}[C(F_0^{(\hat{A})}, F_1^{(\hat{A})})] > \mathbb{E}[C(F_0^{(\hat{B})}, F_1^{(\hat{B})})] \tag{66}$$

The above corollary states that for $\Sigma \in \Theta(p, r, k, \tau, \lambda)$, then provided that the Chernoff information of the population version of LOL is larger than the Chernoff information of the population version of PCA, we can choose n sufficiently large (as compared to λ and τ and k) such that the expected Chernoff information for the sample version of LOL is also larger than the expected Chernoff information of the sample version of PCA. We emphasize that it is necessary that the LOL and the PCA version are both

projected into the top $d \leq r$ dimension of the sample covariance matrices. The constants M and c in the statement of the above corollary are chosen so that M (which depends on M_0 and M_1 in the statement of Theorem 3) is sufficiently large and c (which depends on c_0) is sufficiently small to ensure that the bound in Supp. Eq. (65) is sufficiently small. If $C(F_0^{(A)}, F_1^{(A)}) > C(F_0^{(B)}, F_1^{(B)})$ and $\|\hat{V}\hat{V}^\top - VV^\top\|$ is sufficiently small, then $\mathbb{E}[C(F_0^{(\hat{A})}, F_1^{(\hat{A})})] > \mathbb{E}[C(F_0^{(\hat{B})}, F_1^{(\hat{B})})t]$ as desired.

Supplementary Note 5 Real-Data Performance Analysis

PCA, the industry-standard dimensionality reduction technique for high-dimensional problems, is compared to LOL, RP, and rrLDA in terms of cross-validated classification error.

In the experiments, we used k fold cross-validation. Testing sets were rotated across all folds, with the training sets comprising the remaining $k-1$ folds. A low-dimensional projection matrix $\boldsymbol{A}$ is first learned through the training set, and the low-dimensional training points are then used to train an LDA classifier C. The testing points are embedded via $\boldsymbol{A}$, and classification error is determined using the trained classifier C.

Performance is assessed using Cohen's kappa [7], which normalizes the classification error typically between 0 (the classifier performs no better than the classifier which guesses the most-likely class from the training set, the chance classifier) and 1 (the trained classifier performs perfectly). Negative scores can be achieved if the trained classifier performs worse than the chance classifier. The effect size is measured as the difference between Cohen's kappa for the trained classifier after embedding with PCAκ(PCA) and the trained classifier after embedding with technique ε, $\kappa(\varepsilon)$. Table 1 provides details about each neuroimaging dataset.

Problem	Sample Size (n)	Training Size*	# Features (p)	Classes (K)	Source
Templeton114	111	100	$> 1.5 \times 10^8$	2	MRN
BNU1	110	100	$> 1.5 \times 10^8$	2	CoRR [22]
BNU3	47	43	$> 1.5 \times 10^8$	2	CoRR [22]
SWU4	453	407	$> 1.5 \times 10^8$	2	CoRR [22]
KKI2009	42	38	$> 1.5 \times 10^8$	2	KKI [12]
Genomics	340	306	745,184	2	Douville et al. [9]

Supplementary Table 1. Table of datasets used in this study. The top 5 datasets (neuroimaging) are pre-processing by only registering the brains to the MNI152 template[11, 17, 19, 20]. The neuroimaging dataset comprises a total of 5 classification problems (5 datasets across a single sex classification task). The bottom dataset (genomics) is pre-processed by aligning sequencing data to $745,184$ amplicons on the human genome. The genomics dataset comprises two benchmark classification problems (sex or age).

Supplementary Note 6 Extensions to Other Supervised Learning Problems

6.1 Large numbers of classes

Here, we explore an experiment in which the number of classes increases for a given simulation. We look at the multiclass hump-K problem, described in Section Supplementary Note 3. In this simulation, while the space spanned by the differences of means conveys more information than the directions of

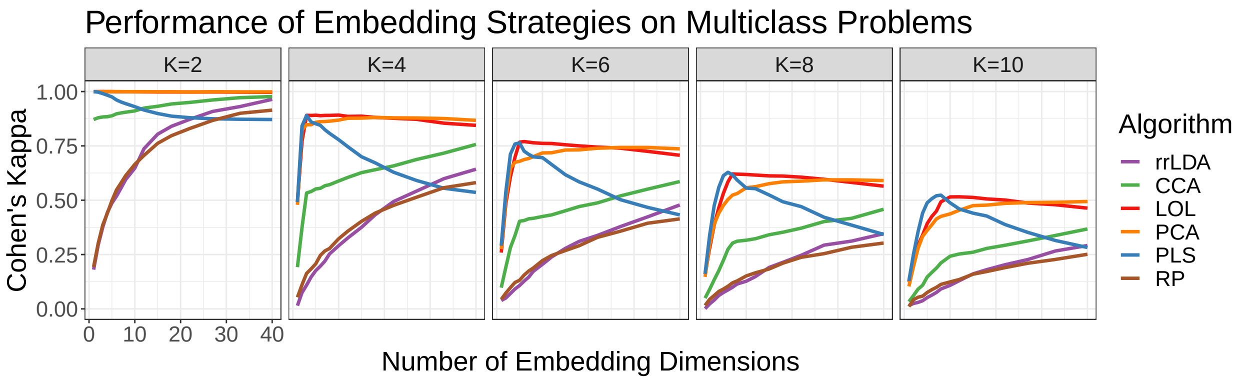

Supplementary Figure 1. The Multiclass Hump Simulation. We show the results of the multiclass trunk problem, as the number of classes increases from 2 to 10, with the number of dimensions and the number of samples fixed. Effect size is measured with Cohen's Kappa. `LOL` and `PLS` provide better performance over competing techniques including `PCA`, and this gap widens as the number of classes increases.

maximal variance, we expect that the shift in the means for a given class at a given dimension should also increase the variance fractionally in that direction as well. Supplementary Figure 1A shows the simulation setup, for $K = 10$. Supplementary Figure 1B indicates the misclassification rate as a function of Cohen's Kappa. We use Cohen's Kappa instead of the misclassification rate for direct evaluation since K varies widely across these simulations at a fixed number of total samples $n = 128$, making the difficulty of the problem as K increases two-fold: not only are there more classes, but there are also fewer examples of each class per simulation setting. In all cases, the best random classifier would be the classifier that continually guesses a single class continuously, which has expected accuracy of $\frac{1}{K}$. On all simulations, we see that both `PLS` and `LOL` rapidly approach a higher Kappa statistic (better performance relative the random classifier) as they learn the space spanned by the differences of means. `PLS` rapidly declines in performance as successive dimensions are added, and `LOL` sees a small performance decline, as successive dimensions should convey no information regarding the class. `PCA` is able to ultimately identify the space spanned by the differences of the means, but takes far more embedding dimensions to do so, and yields a lower Kappa statistic than either of the other two strategies.

6.2 Hypothesis Testing

The utility of incorporating the mean difference vector into supervised machine learning extends beyond classification. In particular, hypothesis testing can be considered as a special case of classification, with a particular loss function. We therefore apply the same idea to a hypothesis testing scenario. The multivariate generalization of the t-test, called Hotelling's Test, suffers from the same problem as does the classification problem; namely, it requires inverting an estimate of the covariance matrix, which would result in a matrix that is low-rank and therefore singular in the high-dimensional setting. To mitigate this issue in the hypothesis testing scenario, prior work applied similar tricks as they have done in the classification setting. One particularly nice and related example is that of Lopes et al. [14], who addresses this dilemma by using random projections to obtain a low-dimensional representation, following by applying Hotelling's Test in the lower-dimensional subspace. Supplementary Figure 2A and B show the power of their test (labeled `RP`) alongside the power of `PCA`, `LOL`, and `LFL` for two different conditions. In each case we use the different approaches to project to low dimensions, followed by using Hotelling's test on the projected data. In the first example the true covariance matrix is diagonal, and in

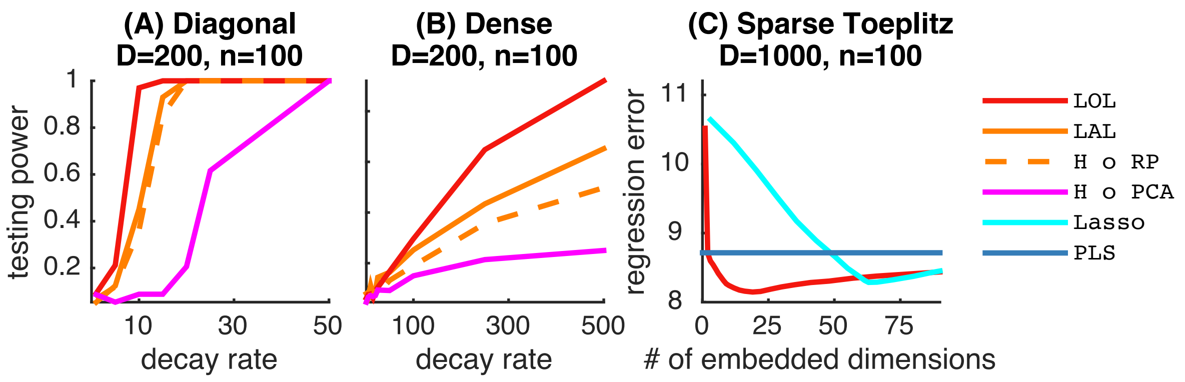

Supplementary Figure 2. The intuition of including the mean difference vector is equally useful for other supervised manifold learning problems, including testing and regression. **(A)** and **(B)** show two different high-dimensional testing settings, as described in Methods. Power is plotted against the decay rate of the spectrum, which approximates the effective number of dimensions. LOL composed with Hotelling's test outperforms the random projections variants described in [14], as well as several other variants. **(C)** A sparse high-dimensional regression setting, as described in Methods, designed for sparse methods to perform well. Log_{10} mean squared error is plotted against the number of projected dimensions. LOL composed with linear regression outperforms Lasso (cyan), the classic sparse regression method, as well as partial least squares (PLS; black). These three simulation settings therefore demonstrate the generality of this technique.

the second, the true covariance matrix is dense. The horizontal axis on both panels characterizes the decay rate of the eigenvalues, so larger numbers imply the data is closer to low-rank (see Methods for details). The results indicate that the LOL test has higher power for essentially all scenarios. Moreover, it is not merely replacing random projections with PCA (solid magenta line), nor simply incorporating the mean difference vector (dashed green line), but rather, it appears that LOL for testing uses both modifications to improve performance.

6.3 Regression

High-dimensional regression is another supervised learning method that can use the LOL idea. Linear regression, like classification and Hotelling's Test, requires inverting a matrix as well. By projecting the data onto a lower-dimensional subspace first, followed by linear regression on the low-dimensional data, we can mitigate the curse of high-dimensions. To choose the projection matrix, we partition the data into K partitions (we select $K = 10$ arbitrarily), based on the percentile of the target variable, we obtain a K-class classification problem. Then, we can apply LOL to learn the projection. Supplementary Figure 2C shows an example of this approach, contrasted with Lasso and partial least squares, in a sparse simulation setting (see Methods for details). LOL is able to find a better low-dimensional projection than Lasso, and performs significantly better than partial least squares, for essentially all choices of number of dimensions to project into.

Supplementary Note 7 The R implementation of LOL

Supplementary Figure 3 shows the R implementation of LOL for binary classification using FlashMatrix [21]. The implementation takes a $D \times I$ matrix, where each column is a training instance and each instance has D features, and outputs a $D \times k$ projection matrix.

```r
LOL <- function(m, labels, k) {
        counts <- fm.table(labels)
        num.labels <- length(counts$val)
        num.features <- dim(m)[1]
        nv <- k - (num.labels - 1)
        gr.sum <- fm.groupby(m, 1, fm.as.factor(labels, 2), fm.bo.add)
        gr.mean <- fm.mapply.row(gr.sum, counts$Freq, fm.bo.div, FALSE)
        diff <- fm.get.cols(gr.mean, 1) - fm.get.cols(gr.mean, 2)
        svd <- fm.svd(m, nv=0, nu=nv)
        fm.cbind(diff, svd$u)
}
```

Supplementary Figure 3. The R implementation of LOL.

Pseudocode 1 Simple pseudocode for two class LOL on sample data.

Input: X a $p \times n$ matrix ($n \ll p$), where columns are observations; rows are features. An n length vector of observation labels, $\mathbf{y}$. An integer k to specify desired output dimension.

Output: $\boldsymbol{A} \in \mathbb{R}^{p \times k}$

function LOL.TRAIN(X, Y, k)
  for all $j \in J$ **do**
    $n_j = \sum_{i=1}^{n} \boldsymbol{I}(y_i = j)$ ▷ sample size per class
    $\hat{\mu}_j = \frac{1}{n_j} \sum_{i=1}^{n} \boldsymbol{x}_i \boldsymbol{I}(y_i = j)$ ▷ class means
  end for
  $\hat{\delta} = \hat{\mu}_1 - \hat{\mu}_2$ ▷ difference of means
  $\hat{\delta} = \hat{\delta} / \left\| \hat{\delta} \right\|$ ▷ unit normalize difference of means
  for all $i \in [n]$ **do**
    $\widetilde{x}_i = \boldsymbol{x}_i - \hat{\mu}_{y_i}$ ▷ class centered data
  end for
  $[\hat{u}, \hat{d}, \hat{v}] = \texttt{svds}(\widetilde{x}, k-1)$ ▷ compute top k singular vectors
  $A = [\hat{\delta}, \hat{u}]$ ▷ concatenate difference of the means and the top k right singular vectors
end function

Supplementary References

[1] J. Ahn and J. S. Marron. The maximum data piling direction for discrimination. *Biometrika*, 97: 254–259, 2010. 7

[2] E. Anderson, Z. Bai, C. Bischof, S. Blackford, J. Demmel, J. Dongarra, J. Du Croz, A. Greenbaum, S. Hammerling, A. McKenney, and D. Sorensen. *LAPACK Users' Guide: Third Edition*. SIAM, 1999. ISBN 0898714478. URL https://books.google.com/books?hl=en&lr=&id=AZlvEnr9gCgC&pgis=1. 4

[3] L. Breiman. Statistical modeling: The two cultures. *Statistical Science*, 16(3):199–231, 2001. 5

[4] Tony Cai, Zongming Ma, and Yihong Wu. Optimal estimation and rank detection for sparse spiked covariance matrices. *Probab. Theory Related Fields*, 161(3-4):781–815, apr 2015. 17

[5] William R. Carson, Minhua Chen, Miguel R. D. Rodrigues, Robert Calderbank, and Lawrence Carin. Communications-Inspired Projection Design with Application to Compressive Sensing. *arXiv*, Jun 2012. doi: 10.1137/120878380. 2

[6] H. Chernoff. A measure of asymptotic efficiency for tests of a hypothesis based on the sum of observations. *Annals of Mathematical Statistics*, 23:493–507, 1952. 10

[7] Jacob Cohen. A coefficient of agreement for nominal scales. *Educational and Psychological Measurement*, 20(1):37–46, 1960. doi: 10.1177/001316446002000104. URL https://doi.org/10.1177/001316446002000104. 18

[8] I. Csizár. Information-type measures of difference of probability distributions and indirect observations. *Studia Scientiarum Mathematicarum Hungarica*, 2:229–318, 1967. 11

[9] Christopher Douville, Joshua D. Cohen, Janine Ptak, Maria Popoli, Joy Schaefer, Natalie Silliman, Lisa Dobbyn, Robert E. Schoen, Jeanne Tie, Peter Gibbs, Michael Goggins, Christopher L. Wolfgang, Tian-Li Wang, Ie-Ming Shih, Rachel Karchin, Anne Marie Lennon, Ralph H. Hruban, Cristian Tomasetti, Chetan Bettegowda, Kenneth W. Kinzler, Nickolas Papadopoulos, and Bert Vogelstein. Assessing aneuploidy with repetitive element sequencing. *Proc. Natl. Acad. Sci. U.S.A.*, 117(9): 4858–4863, Mar 2020. ISSN 0027-8424. doi: 10.1073/pnas.1910041117. 18

[10] Jianqing Fan, Yang Feng, and Xin Tong. A road to classification in high dimensional space: the regularized optimal affine discriminant. *Journal of the Royal Statistical Society: Series B (Statistical Methodology)*, 74(4):745–771, September 2012. ISSN 13697412. doi: 10.1111/j.1467-9868.2012.01029.x. URL http://doi.wiley.com/10.1111/j.1467-9868.2012.01029.x. 2

[11] Mark Jenkinson et al. FSL. *NeuroImage*, 62(2):782–90, aug 2012. ISSN 1095-9572. URL http://www.ncbi.nlm.nih.gov/pubmed/21979382. 18

[12] Bennett A Landman, Alan J Huang, Aliya Gifford, Deepti S Vikram, Issel Anne L Lim, Jonathan A D Farrell, John A Bogovic, Jun Hua, Min Chen, Samson Jarso, Seth A Smith, Suresh Joel, Susumu Mori, James J Pekar, Peter B Barker, Jerry L Prince, and Peter C M van Zijl. Multi-parametric neuroimaging reproducibility: a 3-T resource study. *Neuroimage*, 54(4):2854–2866, February 2011. 18

[13] C. C. Leang and D. H. Johnson. On the asymptotics of M-hypothesis bayesian detection. *IEEE Transactions on Information Theory*, 43:280–282, 1997. 11

[14] Miles Lopes, Laurent Jacob, and Martin J. Wainwright. A More Powerful Two-Sample Test in High Dimensions using Random Projection. In *Neural Information Processing Systems*, pages 1206–1214, 2011. URL http://papers.nips.cc/paper/4260-a-more-powerful-two-sample-test-in-high-dimensions-using-random-projectio 19, 20

[15] Qing Mai, Hui Zou, and Ming Yuan. A direct approach to sparse discriminant analysis in ultra-high dimensions. *Biometrika*, 99(1), Feb 2012. doi: 10.2307/41720670. 2

[16] K. V. Mardia, J. T. Kent, and J. M. Bibby. *Multivariate Analysis*. Academic Press, 1979. 7

[17] John Mazziotta et al. A four-dimensional probabilistic atlas of the human brain. *Journal of the American Medical Informatics Association*, 8(5):401–430, 2001. 18

[18] H. Shin and R. L. Eubank. Unit canonical correlations and high-dimensional discriminant analysis. *Journal of Statistical Computation and Simulation*, 81:167–178, 2011. 7

[19] Stephen M Smith et al. Advances in functional and structural MR image analysis and implementation as FSL. *NeuroImage*, 23 Suppl 1:S208–19, jan 2004. ISSN 1053-8119. URL http://www.ncbi.nlm.nih.gov/pubmed/15501092. 18

[20] Mark W Woolrich et al. Bayesian analysis of neuroimaging data in FSL. *NeuroImage*, 45(1 Suppl): S173–86, mar 2009. ISSN 1095-9572. URL http://www.sciencedirect.com/science/article/pii/S1053811908012044. 18

[21] Da Zheng, Disa Mhembere, Joshua T Vogelstein, Carey E Priebe, and Randal Burns. Flashmatrix: Parallel, scalable data analysis with generalized matrix operations using commodity ssds. *arXiv preprint arXiv:1604.06414*, 2016. 20

[22] Xi-Nian Zuo, Jeffrey S Anderson, Pierre Bellec, Rasmus M Birn, Bharat B Biswal, Janusch Blautzik, John CS Breitner, Randy L Buckner, Vince D Calhoun, F Xavier Castellanos, et al. An open science resource for establishing reliability and reproducibility in functional connectomics. *Scientific data*, 1:140049, 2014. 18
